# Back to Tanganyika: a case of recent trans-species-flock dispersal in East African haplochromine cichlid fishes

**DOI:** 10.1098/rsos.140498

**Published:** 2015-03-04

**Authors:** Britta S. Meyer, Adrian Indermaur, Xenia Ehrensperger, Bernd Egger, Gaspard Banyankimbona, Jos Snoeks, Walter Salzburger

**Affiliations:** 1 Zoological Institute, University of Basel, Basel 4051, Switzerland; 2 Department of Biology, University of Burundi, Bujumbura, Burundi; 3 Royal Museum for Central Africa, Tervuren 3080, Belgium; 4 Laboratory of Biodiversity and Evolutionary Genomics, University of Leuven, Leuven 3000, Belgium

**Keywords:** *Haplochromis* sp. ‘Chipwa’, adaptive radiation, superflock, Lake Victoria

## Abstract

The species flocks of cichlid fishes in the East African Great Lakes are the largest vertebrate adaptive radiations in the world and illustrious textbook examples of convergent evolution between independent species assemblages. Although recent studies suggest some degrees of genetic exchange between riverine taxa and the lake faunas, not a single cichlid species is known from Lakes Tanganyika, Malawi and Victoria that is derived from the radiation associated with another of these lakes. Here, we report the discovery of a haplochromine cichlid species in Lake Tanganyika, which belongs genetically to the species flock of haplochromines of the Lake Victoria region. The new species colonized Lake Tanganyika only recently, suggesting that faunal exchange across watersheds and, hence, between isolated ichthyofaunas, is more common than previously thought.

## 2. Introduction

Adaptive radiation, the rapid evolution of novel species as a consequence of adaptation to distinct ecological niches, is thought to have played an important role in the origin of phenotypic diversity [1]. The species flocks of cichlid fishes in the African Great Lakes; Tanganyika, Malawi and Victoria are the most species-rich vertebrate adaptive radiations, consisting of hundreds of endemic species each [2–4]. Lake Tanganyika, the oldest lake, harbours the genetically and phenotypically most diverse cichlid assemblage comprising 12–16 ‘tribes’ [5]. The radiations in Lakes Malawi and Victoria involve only one of these tribes, the Haplochromini, making this the most species-rich cichlid lineage [4].

The haplochromines probably originated in the area of Lake Tanganyika, from where they colonized water bodies in large parts of Africa, including Lakes Malawi and Victoria [6–8]. This ‘out of Tanganyika’ scenario [6] implies that the seeding events of the haplochromine radiations in Lakes Malawi and Victoria date back to 1–5 and less than 0.25 Ma, respectively [6–9]. The latter radiation is not confined to only the basin of Lake Victoria, but includes the cichlid faunas of other lakes and rivers in the area, including Lakes Edward, George, Kivu and the Lake Rukwa drainage; it is hence referred to as the ‘Lake Victoria region superflock’ (LVRS) [6,7,10].

While Lake Tanganyika's cichlid assemblage has long been regarded as polyphyletic [11], the haplochromines from Lake Malawi and the LVRS were considered reciprocally monophyletic [7,12,13]. This view has recently been challenged with the analysis of large sets of nuclear DNA markers, which uncovered a polyphyletic origin of Lake Malawi's haplochromines [14,15], and high levels of shared genetic polymorphisms between the cichlid faunas of all three lakes [15,16]. These findings, together with the identification of similar or even identical genotypes across large geographical scales [17,18], suggest that the hydrologic systems in East Africa are more permeable for cichlids than previously thought. It has even been proposed that riverine species have ‘transported’ polymorphisms between lakes [15].

Interestingly, however, not a single case of a recent colonization of a Great Lake through a riverine lineage has been documented, and none of these lakes is known to contain a species belonging to a lineage associated with another Great Lake's radiation. Here we report the discovery of a haplochromine cichlid species in Lake Tanganyika, which belongs genetically to the LVRS.

## 3. Material and methods

**Figure 1.**
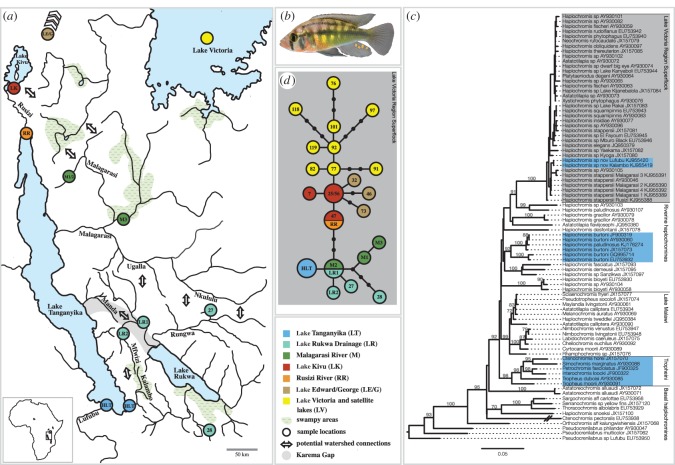
(*a*) Map of the study area indicating sample locations and potential watershed connections. (*b*) *Haplochromis* sp. ‘Chipwa’ (male) from LT. (*c*) ML phylogeny of haplochromine cichlids based on the mitochondrial ND2. *Haplochromis* sp. ‘Chipwa’ is firmly placed within the LVRS (grey box); the specimens from LT are depicted in blue. (*d*) Mitochondrial haplotype genealogy of representative haplotypes of the LVRS and the new species (see also the electronic supplementary material, figure S3) based on a 365 bp segment of the control region. The identification of a shared haplotype between the Malagarasi and the LR basin (M2/LR1) corroborates a recent connection between these watersheds, e.g. via ‘Ugalla–Rungwa’ or ‘Nkululu–Rungwa’ connections [19]. Colour-codes correspond to (*a*) and (*c*), haplotype numbers refer to [7].

In 2011 and 2012, we collected 12 specimens of a new haplochromine species (named *Haplochromis* sp. ‘Chipwa’ hereafter) in a shoreline habitat within Lake Tanganyika at Chipwa Village, between 500 and 1000 m south from the Kalambo River mouth. Five additional specimens were sampled in 2011 in the Lufubu River delta on Lake Tanganyika's western shoreline (open water distance between these locations: more than 55 km; figure 1*a*,*b*). In both localities, the new species co-occurs with the widespread haplochromine *Astatotilapia burtoni* found within Lake Tanganyika and in affluent rivers [20]. The new taxon was identified as undescribed species in the field by A.I.

For comparative reasons, we sampled additional haplochromines, including a morphologically similar species (*Haplochromis stappersii*) from rivers Malagarasi (*n*=4) and Rusizi (*n*=1) (electronic supplementary material, tables S1–S3). Sampling was performed using our standard operating procedure [21]; vouchers were deposited at the University of Basel or the Royal Museum of Central Africa, Tervuren.

In order to place the new taxon into a phylogenetic context, we amplified and sequenced two nuclear (*ednrb1*: 524 bp; *phpt1*: 434 bp) and two mitochondrial (mtDNA) loci (d-loop: 373 bp; ND2: 1047 bp), following the protocols described elsewhere [21,22]. These markers were chosen on the basis of the existence of large quantities of reference data on GenBank. The newly obtained sequences were inspected by eye in CodonCodeAligner, combined with available data from GenBank, aligned with Mafft [23], and the appropriate models of molecular evolution were determined with jMligner, combined with available data from GenBank, aligned with Mafft [23], and the appropriate models of molecular evolution were determined with odeltest [24]. All specimens of the new species were identical in all four loci.

To identify the placement of the new species in the haplochromine phylogeny, we performed a step-wise approach using three different datasets: first, we wanted to confirm our *ad hoc* assumption that the new taxon does not belong to any of the Tanganyikan cichlid lineages (and genera) known to date. To this end, we combined the nuclear and ND2 sequences of the new species with a representative set including all East African cichlid lineages [21], resulting in a total of 83 taxa. The concatenated data (2001 bp) was analysed using Bayesian inference with MrBayes [25] (10 000 000 generations, four chains, two runs, 25% burn-in, three partitions: GTR+I+*Γ*; GTR+I+*Γ*; GTR+*Γ*) and maximum likelihood (ML) with GARLI (http://garli.nescent.org) (50 runs, 500 bootstrap replicates; three partitions: TIM3+I+*Γ*; TVM+I+*Γ*; TPM2uf+*Γ*). In a second step, we focused on ND2 only, as many more reference data are available for this common marker in cichlids [6,8]. We again combined our data with available sequences from GenBank (216 taxa in total) and used MrBayes (3 000 000 generations, four chains, two runs, 25% burn-in; GTR+I+*Γ*) and GARLI (50 runs, 500 bootstraps; TIM2+I+*Γ*). On the basis of this tree, we selected 86 taxa for an in-depth analysis focusing on the species belonging to the LVRS and its closest sister taxa (MrBayes: 10 000 000 generations, four chains, two runs, 25% burn-in, GTR+I+*Γ*; GARLI: 50 runs, 500 bootstraps, TrN+I+*Γ*). Finally, we integrated the mitochondrial control region sequences of *H*. sp. ‘Chipwa’ in the largest available dataset of members of the LVRS [7]. We performed an analysis using 178 unique mitochondrial haplotypes [7], representing about 900 specimens of the LVRS plus outgroup taxa, using GARLI (50 runs; 500 bootstraps; K81uf+I+*Γ*). On the basis of the resultant tree, we chose a representative subset of 27 sequences to construct a haplotype genealogy following the method described in [19] and using the first segment of the mitochondrial control region (373 bp).

## 4. Results

The analysis of the concatenated nuclear and mtDNA dataset resulted in highly congruent trees (electronic supplementary material, figure S1), in which *H*. sp. ‘Chipwa’ formed a strongly supported clade with four taxa representing the LVRS (ML bootstrap = 100, posterior probability = 1), thus confirming previous results based on a large set of nuclear DNA markers [26].

In the more inclusive ND2 phylogeny, the new species was firmly placed within the LVRS *sensu* [7] (electronic supplementary material, figure S2; ML bootstrap = 100, posterior probability = 1). Within this clade, the single ND2 haplotype of the new species from Lake Tanganyika clustered with *H. stappersii* from the Malagarasi River plus another undescribed species from Tanzania (figure 1*c*). Interestingly, two *H. stappersii* were not part of this clade: the sample from Rusizi River in Burundi and the one with unknown sampling location used by Schwartzer *et al*. [18], suggesting that specimens previously identified as*H. stappersii* are not reciprocally monophyletic and belong to at least two distinct mitochondrial lineages.

In the mtDNA haplotype genealogy, the new species was grouped into a clade of riverine taxa derived from the central haplotype of the LVRS (haplotype 25 in [7]; see the electronic supplementary material, figure S3). The reduced dataset (figure 1*d*) highlights that the single haplotype found in *H*. sp. ‘Chipwa’ from Lake Tanganyika is derived from the central haplotype of this riverine clade (M2/LR1) by one mutation (nucleotide divergence: 0.29%). We refrained from performing a molecular clock analysis here, which is problematic with just one mutational difference. However, a single difference in the cichlids' mitochondrial control region is typically interpreted as recent and in the range of a maximum of tens of thousands of years [7,9].

## 5. Discussion

In this study, we report the discovery of a haplochromine species in Lake Tanganyika, which belongs to a clade of riverine haplochromines that is part of the LVRS (figure 1; electronic supplementary material, figures S1–S3). The phylogenetic position of the new species and the existence of identical mtDNA haplotypes on both sides of Lake Tanganyika suggest that this taxon colonized this lake recently and spread across its southern basin. Accidental translocation, e.g. with aquacultured tilapia, seems unlikely given the absence of farmed tilapia at the sampling localities. Instead, it appears likely that the new species entered Lake Tanganyika naturally.

East Africa is a geologically active area and it has been assumed that river captures mediated by tectonic movements, erosion and fluctuations in precipitation allowed for past connections between watersheds [27–30]. Since the mtDNA haplotype of the new species (HLT in figure 1) is derived from the central haplotype (M2/LR1) found in the Malagarasi and in the Lake Rukwa drainage, two alternative dispersal scenarios emerge: either via the Malagarasi River followed by southward coastal migration, or from the Lake Rukwa drainage. Given the large geographical distance between the Malagarasi River and the collection sites and that we never caught any specimen in the coastline north of the Kalambo estuary, the latter scenario appears more plausible—especially, since geological evidence suggests that Lake Rukwa was connected to Lake Tanganyika in the Early Holocene via the Karema Gap [29]. The existence of such a connection has further been corroborated with fossil molluscs and ostracods in Lake Rukwa, which resemble extant taxa from Lake Tanganyika [28]. Another recent Lake Rukwa–Lake Tanganyika connection has been hypothesized in the Kalambo-Mwimbi fault, where rivers Kalambo and Mfiwizi run, in close proximity and in opposite direction, through a swampy depression [27]. Any fish migrating downstream the Kalambo River would, however, face the challenge of a 221 m high waterfall.

With the finding of a member of the LVRS in Lake Tanganyika, we provide, to our knowledge, the first record of a cichlid species in an East African Great Lake that features genetic affinities to the fauna of another Great Lake. More precisely, we show that a haplochromine species belonging to the most recent large-scale cichlid adaptive radiation, the LVRS dated at less than 0.25 Ma [6–9], managed to migrate into the much older Lake Tanganyika, and to establish itself alongside the existing lake endemics. *Haplochromis* sp. ‘Chipwa’ thus represents yet another cichlid lineage that independently colonized Lake Tanganyika. Our discovery thus lends empirical support to the hypothesis that occasional migration of riverine taxa into lakes might have ‘transported’ genetic polymorphism between the cichlid species flocks in the East African Great Lakes [15]. Note, however, that we only demonstrated the first step required by the ‘transporter hypothesis’, i.e. the arrival of a distantly related haplochromine species into an established cichlid radiation. Whether this resulted in the second step, i.e. gene-flow from a divergent lineage into an established lacustrine species, remains unanswered and should be examined in the future.

Taken together, we demonstrate that recent faunal exchange occurred between the otherwise non-overlapping cichlid assemblages of the LVRS and Lake Tanganyika, thereby extending the area covered by LVRS taxa to now also include the southern part of Lake Tanganyika and affluent rivers. Our findings are in line with recent reports of shared mtDNA haplotypes across large geographical scales in haplochromines [17,18] and, particularly, with the view that faunal exchange between cichlid faunas of rivers and lakes is more common than previously thought [15]. We thus suggest that more attention should be directed towards the survey of riverine cichlid communities, which are understudied compared to the endemic faunas of Lakes Tanganyika, Malawi and Victoria.

## Supplementary Material

Table_S1_acc.pdf

## Supplementary Material

Table_S2_acc.pdf

## Supplementary Material

Table_S3_acc.pdf

## Supplementary Material

S1.pdf

## Supplementary Material

S2.pdf

## Supplementary Material

S3.pdf

## Data accessibility

Sequence data has been deposited at GenBank under the accession numbers KJ955381-KJ955446.

## References

[1] Schluter D 2000 The ecology of adaptive radiation. Oxford, UK: Oxford University Press.

[2] Kocher TD 2004 Adaptive evolution and explosive speciation: the cichlid fish model. Nat. Rev. Genet. 5, 288–298. (doi:10.1038/nrg1316) 15131652 10.1038/nrg1316

[3] Seehausen O 2006 African cichlid fish: a model system in adaptive radiation research. Proc. R. Soc. B 273, 1987–1998. (doi:10.1098/rspb.2006.3539) 10.1098/rspb.2006.3539 PMC1635482 16846905

[4] Salzburger W 2009 The interaction of sexually and naturally selected traits in the adaptive radiations of cichlid fishes. Mol. Ecol. 18, 169–185. (doi:10.1111/j.1365-294X.2008.03981.x) 18992003 10.1111/j.1365-294X.2008.03981.x

[5] Koblmüller S, Sefc KM, Sturmbauer C 2008 The Lake Tanganyika cichlid species assemblage: recent advances in molecular phylogenetics. Hydrobiologia 615, 5–20. (doi:10.1007/s10750-008-9552-4)

[6] Salzburger W, Mack T, Verheyen E, Meyer A 2005 Out of Tanganyika: genesis, explosive speciation, key-innovations and phylogeography of the haplochromine cichlid fishes. BMC Evol. Biol. 5, 17 (doi:10.1186/1471-2148-5-17) 15723698 10.1186/1471-2148-5-17 PMC554777

[7] Verheyen E, Salzburger W, Snoeks J, Meyer A 2003 Origin of the superflock of cichlid fishes from Lake Victoria, East Africa. Science 300, 325–329. (doi:10.1126/science.1080699) 12649486 10.1126/science.1080699

[8] Koblmüller S, Schliewen UK, Duftner N, Sefc KM, Katongo C, Sturmbauer C 2008 Age and spread of the haplochromine cichlid fishes in Africa. Mol. Phylogenet. Evol. 49, 153–169. (doi:10.1016/j.ympev.2008.05.045) 18582582 10.1016/j.ympev.2008.05.045

[9] Genner MJ, Seehausen O, Lunt DH, Joyce DA, Shaw PW, Carvalho GR, Turner GF 2007 Age of cichlids: new dates for ancient lake fish radiations. Mol. Biol. Evol. 24, 1269–1282. (doi:10.1093/molbev/msm050) 17369195 10.1093/molbev/msm050

[10] Nagl S, Tichy H, Mayer WE, Takezaki N, Takahata N, Klein J 2000 The origin and age of haplochromine fishes in Lake Victoria, East Africa. Proc. R. Soc. Lond. B 267, 1049–1061. (doi:10.1098/rspb.2000.1109) 10.1098/rspb.2000.1109 PMC1690633 10874756

[11] Salzburger W, Meyer A, Baric S, Verheyen E, Sturmbauer C 2002 Phylogeny of the Lake Tanganyika cichlid species flock and its relationship to the Central and East African haplochromine cichlid fish faunas. Syst. Biol. 51, 113–135. (doi:10.1080/106351502753475907) 11943095 10.1080/106351502753475907

[12] Meyer A, Kocher TD, Basasibwaki P, Wilson AC 1990 Monophyletic origin of Lake Victoria cichlid fishes suggested by mitochondrial DNA sequences. Nature 347, 550–553. (doi:10.1038/347550a0) 2215680 10.1038/347550a0

[13] Moran P, Kornfield I, Reinthal PN 1994 Molecular systematics and radiation of the haplochromine cichlids (Teleostei: Perciformes) of Lake Malawi. Copeia 2, 274–288. (doi:10.2307/1446977)

[14] Joyce DA, Lunt DH, Genner MJ, Turner GF, Bills R, Seehausen O 2011 Repeated colonization and hybridization in Lake Malawi cichlids. Curr. Biol. 21, 108 (doi:10.1016/j.cub.2010.11.029) 10.1016/j.cub.2010.11.029 21300271

[15] Loh YH 2013 Origins of shared genetic variation in African cichlids. Mol. Biol. Evol. 30, 906–917. (doi:10.1093/molbev/mss326) 23275489 10.1093/molbev/mss326 PMC3603313

[16] Brawand D 2014 The genomic substrate for adaptive radiation in African cichlid fish. Nature 513, 375–381. (doi:10.1038/nature13726) 25186727 10.1038/nature13726 PMC4353498

[17] Hermann CM, Sefc KM, Koblmüller S 2011 Ancient origin and recent divergence of a haplochromine cichlid lineage from isolated water bodies in the East African Rift System. J. Fish Biol. 79, 1356–1369. (doi:10.1111/j.1095-8649.2011.03101.x) 22026612 10.1111/j.1095-8649.2011.03101.x

[18] Schwartzer J, Swartz ER, Vreven E, Snoeks J, Cotterill FP, Misof B, Schliewen UK 2012 Repeated trans-watershed hybridization among haplochromine cichlids (Cichlidae) was triggered by Neogene landscape evolution. Proc. R. Soc. B 279, 4389–4398. (doi:10.1098/rspb.2012.1667) 10.1098/rspb.2012.1667 PMC3479809 22951733

[19] Salzburger W, Ewing GB, Von Haeseler A 2011 The performance of phylogenetic algorithms in estimating haplotype genealogies with migration. Mol. Ecol. 20, 1952–1963. (doi:10.1111/j.1365-294X.2011.05066.x) 21457168 10.1111/j.1365-294X.2011.05066.x

[20] Theis A, Ronco R, Indermaur A, Salzburger W, Egger B 2014 Adaptive divergence between lake and stream populations of an East African cichlid fish. Mol. Ecol. 23, 5304–5322. (doi:10.1111/mec.12939) 25256664 10.1111/mec.12939

[21] Muschick M, Indermaur A, Salzburger W 2012 Convergent evolution within an adaptive radiation of cichlid fishes. Curr. Biol. 22, 2362–2368. (doi:10.1016/j.cub.2012.10.048) 23159601 10.1016/j.cub.2012.10.048

[22] Meyer BS, Salzburger W 2012 A novel primer set for multilocus phylogenetic inference in East African cichlid fishes. Mol. Ecol. Res. 12, 1097–1104. (doi:10.1111/j.1755-0998.2012.03169.x) 10.1111/j.1755-0998.2012.03169.x 22816488

[23] Katoh K, Standley DM 2013 MAFFT multiple sequence alignment software version 7: improvements in performance and usability. Mol. Biol. Evol. 30, 772–780. (doi:10.1093/molbev/mst010) 23329690 10.1093/molbev/mst010 PMC3603318

[24] Posada D 2008 jModelTest: phylogenetic model averaging. Mol. Biol. Evol. 25, 1253–1256. (doi:10.1093/molbev/msn083) 18397919 10.1093/molbev/msn083

[25] Ronquist F 2012 MrBayes 3.2: efficient Bayesian phylogenetic inference and model choice across a large model space. Syst. Biol. 61, 539–542. (doi:10.1093/sysbio/sys029) 22357727 10.1093/sysbio/sys029 PMC3329765

[26] Meyer BS, Matschiner M, Salzburger W 2015 A tribal level phylogeny of Lake Tanganyika cichlid fishes based on a genomic multi-marker approach. Mol. Phyl. Evol. 83C, 56–71. (doi:10.1016/j.ympev2014.10.009) 10.1016/j.ympev.2014.10.009 PMC4334724 25433288

[27] Seegers L 1996 The fishes of the Lake Rukwa drainage. Ann. Mus. R. Afr. Centr. Sci. Zool. 287, 1–407

[28] Cohen AS, Van Bocxlaer B, Todd JA, McGlue M, Michel E, Nkotagu HH, Grove AT, Delvaux D 2013 Quanternary ostracods and molluscs from the Rukwa Basin (Tanzania) and their evolutionary and palaeobiogeographic implications. Palaeogeogr. Palaeoclimatol. Palaeoecol. 392, 97–97. (doi:10.1016/j.palaeo.2013.09.007)

[29] Delvaux D, Kervyn F, Vittori E, Kajara RSA, Kilembe E 1998 Late Quaternary tectonic activity and lake level change in the Rukwa Rift Basin. J. Afr. Earth Sci. 26, 397–421. (doi:10.1016/S0899-5362(98)00023-2)

[30] Salzburger W, Van Bocxlaer B, Cohen AS 2014 Ecology and evolution of the African Great Lakes and their faunas. Annu. Rev. Ecol. Evol. Syst. 45, 519–545. (doi:10.1146/annurev-ecolsys-120213-091804)

