# Supplementary material for: Back to Tanganyika: a case of recent trans-species-flock dispersal in East African haplochromine cichlid fishes
Source: R Soc Open Sci. 2015 Mar 4;2(3):140498. doi: 10.1098/rsos.140498 (PMC4448823; doi:10.1098/rsos.140498)
Supplement: Table_S1_acc.pdf [file rsos140498supp1.pdf]

Supplementary table 1: List of 83 cichlid specimens, their mitochondrial ND2 and their nuclear gene (ednrb, phpt1) accession numbers and their sample locations.

	GenBank accession numbers			Sampling information	
**Species**	**nd2**	**ednrb**	**phpt1**	**Locality**	**Coordinates**
Altolamprologus calvus	EF462256	JF900248	JF900177	Lake Tanganyika	-
Altolamprologus compressiceps	EF462257	JF900249	JF900178	Lake Tanganyika	-
Asprotilapia leptura	KJ955424	JF900251	JF900180	Lake Tanganyika	-
Haplochromis sp. nov. "Kalambo"	KJ955419	KJ955401	KJ955436	Kalambo River, Zambia	S08°36'06.34"; E031°11'12.73"
Haplochromis sp. nov. "Lufubu"	KJ955420	KJ955402	KJ955437	Lufubu River, Zambia	S08°33'41.25"; E030°43' 26.54"
Astatoreochromis alluaudi	KJ955410	KJ955393	KJ955429	Aquaria Stock, Lake Victoria	-
Astatotilapia burtoni	KJ955411	KJ955394	KJ955430	Aquaria Stock, Lake Tanganyika	-
Astatotilapia burtoni	JF900319	JF900252	JF900181	Lake Tanganyika	-
Astatotilapia calliptera	KJ955412	KJ955398	KJ955431	Aquaria Stock, Lake Malawi	-
Aulonocranus dewindti	AY337782	JF900253	JF900182	Lake Tanganyika	-
Baileychromis centropomoides	KJ955423	KJ955406	KJ955432	Mpulungu Market, Zambia	S8° 45' 56.737" E31° 6' 49.715"
Bathybates graueri	AY663726	JF900254	JF900183	Lake Tanganyika	-
Bathybates vittatus	AY663728	JF900255	JF900184	Lake Tanganyika	-
Benthochromis tricoti	AF317264	JF900256	JF900185	Lake Tanganyika	-
Boulengerochromis microlepis	AF317229	JF900257	JF900186	Lake Tanganyika	-
Callochromis macrops	AY337795	JF900258	JF900187	Lake Tanganyika	-
Chalinochromis brichardi	EF679241	JF900259	JF900188	Lake Tanganyika	-
Cyphotilapia gibberosa	EF679242	JF900260	JF900189	Lake Tanganyika	-
Ctenochromis horei	EU753935	JF900262	JF900191	Lake Tanganyika	-
Cyathopharynx furcifer	AY337781	JF900263	JF900192	Lake Tanganyika	-
Cyprichromis leptosoma	AY740337	JF900264	JF900193	Lake Tanganyika	-
Ectodus descampsii	AY337790	JF900265	JF900195	Lake Tanganyika	-
Enantiopus melanogenys	AY682517	JF900266	JF900194	Lake Tanganyika	-
Eretmodus cyanostictus	AF398220	JF900267	JF900196	Lake Tanganyika	-
Gnathochromis permaxillaris	JF900321	JF900268	JF900197	Lake Tanganyika	-
Gnathochromis pfefferi	U07248	JF900269	JF900198	Lake Tanganyika	-
Grammatotria lemairii	AY337787	JF900270	JF900199	Lake Tanganyika	-
Greenwoodochromis christyi	AY682528	JF900272	JF900201	Lake Tanganyika	-
Haplotaxodon microlepis	EF437497	JF900273	JF900202	Lake Tanganyika	-
Haplochromis obliquidens	KJ955416	KJ955403	KJ955433	Aquaria Stock, Lake Victoria	-
Haplochromis rockkribensis	KJ955418	KJ955404	KJ955434	Aquaria Stock, Lake Victoria	-
Haplotaxodon trifasciatus	EF437492	JF900274	JF900203	Lake Tanganyika	-
Interochromis loocki	JF900322	JF900303	JF900232	Lake Tanganyika	-
Julidochromis ornatus	EF462229	JF900275	JF900204	Lake Tanganyika	-
Lamprologus callipterus	AF398226	JF900276	JF900205	Lake Tanganyika	-
Lamprologus lemairii	EF462271	JF900277	JF900206	Lake Tanganyika	-
Lamprologus ornatipinnis	EF462260	JF900278	JF900207	Lake Tanganyika	-
Limnochromis abeelei	AY682533	JF900279	JF900208	Lake Tanganyika	-
Lepidiolamprologus attenuatus	EF462274	JF900282	JF900211	Lake Tanganyika	-
Lepidiolamprologus elongatus	EF462268	JF900283	JF900212	Lake Tanganyika	-
Lepidiolamprologus cf. profundicola	EF462276	JF900284	JF900213	Lake Tanganyika	-
Limnotilapia dardennii	GQ995724	JF900285	JF900214	Lake Tanganyika	-
Lobochilotes labiatus	U07254	JX402345	JF900215	Lake Tanganyika	-
Microdontochromis tenuidentatus	AY337784	JF900287	JF900216	Lake Tanganyika	-
Neolamprologus furcifer	EF679252	JF900288	JF900217	Lake Tanganyika	-
Neolamprologus modestus	DQ055012	JF900289	JF900218	Lake Tanganyika	-

Neolamprologus prochilus	EF462248	JF900290	JF900219	Lake Tanganyika	-
Neolamprologus pulcher	EF462244	JF900291	JF900220	Lake Tanganyika	-
Neolamprologus savoryi	HM623796	JF900292	JF900221	Lake Tanganyika	-
Neolamprologus sexfasciatus	HM623828	JF900293	JF900222	Lake Tanganyika	-
Neolamprologus tetracanthus	EF462220	JF900294	JF900223	Lake Tanganyika	-
Ophthalmotilapia ventralis	AY337774	JF900295	JF900224	Lake Tanganyika	-
Oreochromis tanganicae	AF317240	JF900296	JF900225	Lake Tanganyika	-
Paracyprichromis brieni	AY740378	JF900297	JF900226	Lake Tanganyika	-
Perissodus microlepis	AF398222	JF900298	JF900227	Lake Tanganyika	-
Plecodus paradoxus	EF437500	JF900299	JF900228	Lake Tanganyika	-
Petrochromis famula	JF900324	JF900301	JF900230	Lake Tanganyika	-
Petrochromis fasciolatus	JF900325	JF900302	JF900231	Lake Tanganyika	-
Petrochromis macrognathus	AY930068	JF900304	JF900233	Lake Tanganyika	-
Petrochromis polyodon	JF900326	JF900305	JF900234	Lake Tanganyika	-
Pharyngochromis acuticeps	KJ955421	KJ955396	KJ955438	Kafue, Zambia	-
Plecodus straeleni	EF437481	JF900306	JF900235	Lake Tanganyika	-
Pseudosimochromis curvifrons	GQ995777	JF900307	JF900236	Lake Tanganyika	-
Pseudotropheus sp. „acei“	KJ955413	KJ955399	KJ955439	Aquaria Stock, Lake Malawi	-
Pseudocrenilabrus multicolor	KJ955425	KJ955395	KJ955440	Aquaria Stock, Lake Malawi	-
Cynotilapia pulpican	KJ955414	KJ955400	KJ955442	Aquaria Stock, Lake Malawi	-
Pundamilia nyererei	KJ955417	KJ955405	KJ955441	Aquaria Stock, Lake Malawi	-
Reganochromis calliurus	AY682544	JF900308	JF900237	Lake Tanganyika	-
Rhamphochromis sp.	KJ955415	KJ955407	KJ955443	Aquaria Stock, Lake Malawi	-
Sarotherodon sp. "Barombi Mbo"	KJ955426	KJ955407	KJ955435	Barombi Mbo, Cameroon	-
Serranochromis macrocephalus	KJ955422	KJ955397	KJ955444	Kafue, Zambia	S14° 58' 25.315" E25° 55' 14.642"
Simochromis diagramma	AY930087	JF900310	JF900239	Lake Tanganyika	-
Telmatochromis dhonti/temporalis	EF679266	JF900311	JF900240	Lake Tanganyika	-
Oreochromis sp.	KJ955427	KJ955408	KJ955445	Kafue, Zambia	S14° 58' 25.315" E25° 55' 14.642"
Tilapia zillii	KJ955428	KJ955409	KJ955446	Daylan, Turkey	N36° 49' 56.349" E28° 38' 13.746"
Trematocara marginatum	JF900327	JF900312	JF900241	Lake Tanganyika	-
Trematochromis benthicola	JF900320	JF900261	JF900190	Lake Tanganyika	-
Trematocara nigrifrons	JF900328	JF900313	JF900242	Lake Tanganyika	-
Tropheus moorii	AY930093	JF900314	JF900243	Lake Tanganyika	-
Tylochromis polylepis	U07268	JF900315	JF900244	Lake Tanganyika	-
Variabilichromis moorii	DQ055016	JF900316	JF900245	Lake Tanganyika	-
Xenotilapia flavipinnis	AY337794	JF900317	JF900246	Lake Tanganyika	-
Xenotilapia spiloptera	AY337788	JF900318	JF900247	Lake Tanganyika	-
