# Supplementary material for: Back to Tanganyika: a case of recent trans-species-flock dispersal in East African haplochromine cichlid fishes
Source: R Soc Open Sci. 2015 Mar 4;2(3):140498. doi: 10.1098/rsos.140498 (PMC4448823; doi:10.1098/rsos.140498)
Supplement: Table_S2_acc.pdf [file rsos140498supp2.pdf]

Supplementary table 2: List of 218 cichlid specimens and their mitochondrial ND2 sequence accession numbers. Specified are the original publications, their sample information and in which analysis there were used.

Species	Published in	Accession number	Locality	Coordinates	Collected by	Fig1c	FigS2
Haplochromis sp. "Chipwa"	Present study	KJ955419	Kalambo River Delta, Zambia	08°36'6.34"S; 031°11'12.73"E	*ZIUB NS_CH4*	+	+
Haplochromis sp. "Chipwa"	Present study	KJ955420	Lufubu River Delta, Zambia	8°33'41.25"S; 030°43' 26.54"E	*ZIUB NS_LU2*	+	+
Haplochromis stappersii "Malagarasi 1"	Present study	KJ955389	Malagarasi River	03°50'56.9"S; 030°18'01.3"E	*Gaspard Banyankimbona, MRAC1840*	+	+
Haplochromis stappersii "Malagarasi 2"	Present study	KJ955390	Malagarasi River	03°51'25.2"S; 030°17'53.5"E	*Gaspard Banyankimbona, MRAC1847*	+	+
Haplochromis stappersii "Malagarasi 3"	Present study	KJ955391	Muvumu-Nkobokobo	03°53'10.8"S; 030°15'16.1"E	*Gaspard Banyankimbona, MRAC12034*	+	+
Haplochromis stappersii "Malagarasi 4"	Present study	KJ955392	SOSUMO-Amont	03°59'33.8"S; 030°12'52.9"E	*Gaspard Banyankimbona, MRAC12087*	+	+
Haplochromis stappersii "Rusizi"	Present study	KJ955388	Gatumba marsh, Rusizi River	03°20'21,6"S; 029°13'56,9"E	*Gaspard Banyankimbona, MRAC6334*	+	+
Boulengerochromis microlepis	Klett & Meyer 2002	AF317229	n/a	n/a	*n/a*		+
Haplochromis burtoni	Kobelmüller et al. 2010	GQ995714	Kalambo, above falls	n/a	Kobelmüller et al. 2010, 7055	+	
Astatoreochromis alluaudi	Koblmüller et al. 2008	EU753923	Lake Kanyaboli, Kenya	n/a	n/a		+
Chetia brevicauda	Koblmüller et al. 2008	EU753924	Buzi River	n/a	n/a		+
Chetia brevis	Koblmüller et al. 2008	EU753925	Incomati River	n/a	n/a		+
Chetia flaviventris	Koblmüller et al. 2008	EU753926	Limpopo river	n/a	n/a		+
Chetia flaviventris	Koblmüller et al. 2008	EU753927	Limpopo river	n/a	n/a		+
Haplochromini sp. 'Lufubu'	Koblmüller et al. 2008	EU753928	Lufubu river, Zambia	n/a	n/a		+
Thoracochromis albolabris	Koblmüller et al. 2008	EU753929	Cunene River	n/a	n/a	+	+
Haplochromis bloyeti	Koblmüller et al. 2008	EU753930	Nyumba ya Mungu, Tanzania	n/a	n/a	+	+
Thoracochromis brauschi	Koblmüller et al. 2008	EU753931	Lake Fwa, DRC	n/a	n/a		+
Haplochromis burtoni	Koblmüller et al. 2008	EU753932	Kalambo River	n/a	n/a	+	+
Thoracochromis buysi	Koblmüller et al. 2008	EU753933	Cunene River	n/a	n/a		+
Astatotilapia calliptera	Koblmüller et al. 2008	EU753934	Lake Kisiba, Tanzania	n/a	n/a	+	+
Ctenochromis horei	Koblmüller et al. 2008	EU753935	Lake Tanganyika	n/a	n/a		+
Orthochromis machadoi	Koblmüller et al. 2008	EU753936	Cunene River	n/a	n/a		+
Haplochromis oligacanthus	Koblmüller et al. 2008	EU753937	Ngoko River, Congo	n/a	n/a		+
Ctenochromis pectoralis	Koblmüller et al. 2008	EU753938	Nyumba ya Mungu, Tanzania	n/a	n/a	+	+
Ctenochromis pectoralis	Koblmüller et al. 2008	EU753939	Nyumba ya Mungu, Tanzania	n/a	n/a		+
Haplochromis phytophagus	Koblmüller et al. 2008	EU753940	Lake Kenyaboli, Kenia	n/a	n/a	+	+
Haplochromis polli	Koblmüller et al. 2008	EU753941	Lower Congo River	n/a	n/a		+
Haplochromis rudolfianus	Koblmüller et al. 2008	EU753942	Lake Turkana	n/a	n/a	+	+
Haplochromis squamipinnis	Koblmüller et al. 2008	EU753943	Lake Edward Uganda	n/a	n/a	+	+
Haplochromis sp. 'Lake Kanyaboli'	Koblmüller et al. 2008	EU753944	Lake Kenyaboli, Kenia	n/a	n/a	+	+
Haplochromis sp. El Fayoum	Koblmüller et al. 2008	EU753945	El Fayoum Oasis, Egypt	n/a	n/a	+	+
Haplochromis sp. 'Mburo Black'	Koblmüller et al. 2008	EU753946	Lake Mburo, Uganda	n/a	n/a	+	+
Nimbochromis venustus	Koblmüller et al. 2008	EU753947	Lake Malawi	n/a	n/a	+	+
Nimbochromis livingstonii	Koblmüller et al. 2008	EU753948	Lake Malawi	n/a	n/a	+	+
Pharyngochromis acuticeps	Koblmüller et al. 2008	EU753949	Rundu, Namibia	n/a	n/a		+
Pseudocrenilabrus sp. Lufubu	Koblmüller et al. 2008	EU753950	Lufubu river, Zambia	n/a	n/a	+	+
Pseudocrenilabrus sp. Lunzua blue	Koblmüller et al. 2008	EU753951	Lunzua River, Zambia	n/a	n/a		+
Pseudocrenilabrus sp. Mweru orange	Koblmüller et al. 2008	EU753952	Lake Mweru	n/a	n/a		+
Pseudocrenilabrus sp. Olushandja	Koblmüller et al. 2008	EU753953	Cunene River, Olushandjia, Namibia	n/a	n/a		+
Sargochromis coulteri	Koblmüller et al. 2008	EU753954	Cunene River, Olushandjia, Namibia	n/a	n/a		+
Sargochromis coulteri	Koblmüller et al. 2008	EU753955	Olushandja, Namibia	n/a	n/a		+
Sargochromis aff. carlottae SK-2008	Koblmüller et al. 2008	EU753956	Kafue Flats, Zambia	n/a	n/a	+	+
Schwetzochromis neodon	Koblmüller et al. 2008	EU753957	Lake Fwa, Congo	n/a	n/a		+
Serranochromis angusticeps	Koblmüller et al. 2008	EU753958	Cunene River	n/a	n/a		+
Serranochromis angusticeps	Koblmüller et al. 2008	EU753959	Cunene River	n/a	n/a		+
Serranochromis stappersi	Koblmüller et al. 2008	EU753960	Lake Bangwuelu, Zambia	n/a	n/a		+
Serranochromis thumbergi	Koblmüller et al. 2008	EU753961	Lake Bangwuelu, Zambia	n/a	n/a		+
Benthochromis horii	Koblmüller et al. 2008	EU753962	Lake Tanganyika	n/a	n/a		+
Tylochromis polylepis	Kocher et al. 1995	U07268	Fish market, Uvira, Kongo	n/a	n/a		+
Haplochromis burtoni	Muschick et al. 2012	JF900319	Kalambo River, Zambia	n/a	ZIUB	+	+
Trematochromis benthicola	Muschick et al. 2012	JF900320	Lake Tanganyika	n/a	ZIUB		+
Gnathochromis permaxillaris	Muschick et al. 2012	JF900321	Lake Tanganyika	n/a	ZIUB		+
Interochromis loocki	Muschick et al. 2012	JF900322	Lake Tanganyika	n/a	ZIUB	+	+
Petrochromis ephippium	Muschick et al. 2012	JF900323	Lake Tanganyika	n/a	ZIUB		+
Petrochromis famula	Muschick et al. 2012	JF900324	Lake Tanganyika	n/a	ZIUB		+
Petrochromis fasciolatus	Muschick et al. 2012	JF900325	Lake Tanganyika	n/a	ZIUB	+	+
Petrochromis polyodon	Muschick et al. 2012	JF900326	Lake Tanganyika	n/a	ZIUB		+
Trematocara marginatum	Muschick et al. 2012	JF900327	Lake Tanganyika	n/a	ZIUB		+
Trematocara nigrifrons	Muschick et al. 2012	JF900328	Lake Tanganyika	n/a	ZIUB		+
Serranochromis macrocephalus "Cutato"	Musilovà et al. 2013	KC146709	Cutato River, Angola	n/a	Musilovà et al. C71		+
Serranochromis macrocephalus	Musilovà et al. 2013	KC146710	Angola	n/a	Musilovà et al. Z80_2		+
Serranochromis macrocephalus	Musilovà et al. 2013	KC146711	Angola	n/a	Musilovà et al. Z80_1		+
Serranochromis macrocephalus "Cuchi"	Musilovà et al. 2013	KC146712	Cuchi River, Angola	n/a	Musilovà et al. K03		+
Serranochromis macrocephalus "Cuchi"	Musilovà et al. 2013	KC146713	Cuchi River, Angola	n/a	Musilovà et al. K05		+
Serranochromis macrocephalus "Cuchi"	Musilovà et al. 2013	KC146714	Cuchi River, Angola	n/a	Musilovà et al. K07		+
Serranochromis macrocephalus "Cuito"	Musilovà et al. 2013	KC146715	Cuito River, Angola	n/a	Musilovà et al. K16		+
Serranochromis macrocephalus "Cuito"	Musilovà et al. 2013	KC146716	Cuito River, Angola	n/a	Musilovà et al. B51n		+
Serranochromis macrocephalus "Cuito"	Musilovà et al. 2013	KC146717	Cuito River, Angola	n/a	Musilovà et al. Z05		+
Serranochromis macrocephalus "Cuito"	Musilovà et al. 2013	KC146718	Cuito River, Angola	n/a	Musilovà et al. Z09		+
Serranochromis macrocephalus "Lomba"	Musilovà et al. 2013	KC146719	Lomba, Angola	n/a	Musilovà et al. C05n		+
Serranochromis macrocephalus "Lomba"	Musilovà et al. 2013	KC146720	Lomba, Angola	n/a	Musilovà et al. C11n		+
Serranochromis macrocephalus "Lomba"	Musilovà et al. 2013	KC146721	Lomba, Angola	n/a	Musilovà et al. C16n		+
Serranochromis macrocephalus "Lomba"	Musilovà et al. 2013	KC146722	Lomba, Angola	n/a	Musilovà et al. C17n		+
Serranochromis macrocephalus "Lomba"	Musilovà et al. 2013	KC146723	Lomba, Angola	n/a	Musilovà et al. C27n		+
Serranochromis macrocephalus "Cuemba"	Musilovà et al. 2013	KC146724	Cuemba River, Angola	n/a	Musilovà et al. V33		+
Serranochromis macrocephalus "Cuemba"	Musilovà et al. 2013	KC146725	Cuemba River, Angola	n/a	Musilovà et al. V35		+
Serranochromis macrocephalus "Cutato"	Musilovà et al. 2013	KC146726	Cutato River, Angola	n/a	Musilovà et al. C70		+
Haplochromis sp. Luando	Musilovà et al. 2013	KC146727	Luando River, Angola	n/a	Musilovà et al. Z38		+

Haplochromis sp. Luando	Musilovà et al. 2013	KC146728	Luando River, Angola	n/a	Musilovà et al. Z35		+
Haplochromis sp. Lomba	Musilovà et al. 2013	KC146729	Lomba, Angola	n/a	Musilovà et al. C52		+
Haplochromis sp. Lomba	Musilovà et al. 2013	KC146730	Lomba, Angola	n/a	Musilovà et al. C50n		+
Serranochromis sp.	Musilovà et al. 2013	KC146731	Angola	n/a	Musilovà et al. Z81		+
Thoracochromis sp. Huando	Musilovà et al. 2013	KC146732	Huando River, Angola	n/a	Musilovà et al. Z21		+
Tilapia sp.	Musilovà et al. 2013	unpublished			Z85		+
Haplochromis. stappersii	Salzburger et al. 2005	AY930046	Malagarasi River, Tanzania	n/a	L. De Vos (5-6/25/92)	+	+
Pseudocrenilabrus philander	Salzburger et al. 2005	AY930047	Zambezi River, Zambia	n/a	aquarium trade	+	+
Orthochromis uvinzae	Salzburger et al. 2005	AY930048	Malagarasi River, Tanzania	n/a	L. Seegers (TZ94-112b)		+
Orthochromis kasuluensis	Salzburger et al. 2005	AY930049	Tanzania	n/a	L. De Vos (T2-July 94)		+
Orthochromis rugufuensis	Salzburger et al. 2005	AY930050	Rugufu River, Tanzania	n/a	L. Seegers (TZ94-121)		+
Orthochromis rubrolabialis	Salzburger et al. 2005	AY930051	Tanzania	n/a	L. Seegers (TZ94-108)		+
Orthochromis luichensis	Salzburger et al. 2005	AY930052	Mkuti River, Luiche Basin, Tanzania	n/a	L. De Vos (T94/3)		+
Orthochromis mazimeroensis	Salzburger et al. 2005	AY930053	Nanganga, Burundi	n/a	L. De Vos (T1-5/27/93)		+
Orthochromis malagaraziensis	Salzburger et al. 2005	AY930054	Nyarungunga River, Burundi	n/a	L. De Vos (T5-5/28/93)		+
Orthochromis mosoensis	Salzburger et al. 2005	AY930055	Ruisseau Gytinya, Burundi	n/a	L. De Vos (T7-5/28/93)		+
Orthochromis malagaraziensis	Salzburger et al. 2005	AY930056	Nyarungunga River, Burundi	n/a	L. De Vos (7-2/19/93)		+
Orthochromis stormsi	Salzburger et al. 2005	AY930057	Kisangani (Lualaba River), DR Congo	n/a	L. De Vos (5/5/95)		+
Haplochromis bloyeti	Salzburger et al. 2005	AY930058	Lukaware River, Kenia	n/a	L. De Vos (F2A-12/93)	+	+
submitted as Ptyochromis sauvagei Haplochromis fischeri	Salzburger et al. 2005	AY930059	Lake Victoria (Kisumu, Kenya)	n/a	L. De Vos (F2B-12/93)	+	+
Haplochromis burtoni	Salzburger et al. 2005	AY930060	Lake Tanganyika	n/a	L. De Vos (31-02/6/92). T34	+	+
Maylandia livingstonii	Salzburger et al. 2005	AY930061	Lake Malawi	n/a	I. Kornfield	+	+
Haplochromis sp. 'Kisangani'	Salzburger et al. 2005	AY930062	Kisangani, (Lualaba River), DR Congo	n/a	L. De Vos (6/13/95)		+
submitted as Ptyochromis sauvagei Haplochromis fischeri	Salzburger et al. 2005	AY930063	Lake Victoria	n/a	A. Meyer, T44	+	+
Platytaeniodus degeni	Salzburger et al. 2005	AY930064	Lake Victoria	n/a	A. Meyer (Pd1)	+	+
Haplochromis sp. V7	Salzburger et al. 2005	AY930065	Lake Victoria	n/a	A. Meyer (V7-Feb 93)	+	+
Tropheus moorii	Salzburger et al. 2005	AY930066	Lake Tanganyika	n/a	E. Verheyen; T66		+
Tropheus moorii	Salzburger et al. 2005	AY930067	Lake Tanganyika	n/a	E. Verheyen; T67		+
Petrochromis macrognathus	Salzburger et al. 2005	AY930068	Lake Tanganyika	n/a	J. Snoeks, MRAC		+
Melanochromis auratus	Salzburger et al. 2005	AY930069	Lake Malawi	n/a	aquarium	+	+
Pseudocrenilabrus multicolor victoriae	Salzburger et al. 2005	AY930070	Lake Kanyaboli, Kenya	n/a	R. Abila (R082-2002)		+
Astatoreochromis alluaudi	Salzburger et al. 2005	AY930071	Lake Kanyaboli, Kenya	n/a	R. Abila (R101-2002)	+	+
Astatotilapia sp. R184	Salzburger et al. 2005	AY930072	Lake Kanyaboli, Kenya	n/a	R. Abila (R184-2002)	+	+
Astatotilapia sp. R185	Salzburger et al. 2005	AY930073	Lake Kanyaboli, Kenya	n/a	R. Abila (R185-2002)	+	+
Haplochromis sp. 'dwarf big eye'	Salzburger et al. 2005	AY930074	Lake Kanyaboli, Kenya	n/a	R. Abila (R280-2002)	+	+
Astatoreochromis alluaudi	Salzburger et al. 2005	AY930075	Lake Kanyaboli, Kenya	n/a	R. Abila (R281-2002)		+
Xystichromis phytophagus	Salzburger et al. 2005	AY930076	Lake Kanyaboli, Kenya	n/a	R. Abila (R670-2002)	+	+
Haplochromis insidiae	Salzburger et al. 2005	AY930077	Lake Kivu	n/a	E. Verheyen	+	+
Haplochromis gracilior	Salzburger et al. 2005	AY930078	Lake Kivu	n/a	E. Verheyen; K8	+	+
Thoracochromis brauschi	Salzburger et al. 2005	AY930080	Lake Fwa	n/a	R. Paul/E. Schraml (9792)		+
Serranochromis sp. 9793	Salzburger et al. 2005	AY930081	Lake Mweru-Wantipa, Zambia	n/a	T. Reuter / E. Schraml (9793)		+
Haplochromis sp. 9796	Salzburger et al. 2005	AY930082	Lake Mburo, Uganda	n/a	E. Schraml (9796)	+	+
Haplochromis squamipinnis	Salzburger et al. 2005	AY930083	Lake Edward	n/a	E. Schraml (9813)	+	+
Tropheus polli	Salzburger et al. 2005	AY930084	Lake Tanganyika	n/a	E. Verheyen		+
Tropheus duboisi	Salzburger et al. 2005	AY930085	Lake Tanganyika	n/a	E. Verheyen; M7	+	+
Tropheus brichardi	Salzburger et al. 2005	AY930086	Lake Tanganyika	n/a	E. Verheyen; M85		+
Simochromis diagramma	Salzburger et al. 2005	AY930087	Lake Tanganyika	n/a	E. Verheyen		+
Simochromis marginatus	Salzburger et al. 2005	AY930088	Lake Tanganyika	n/a	E. Verheyen	+	+
Cyrtocara moorii	Salzburger et al. 2005	AY930089	Lake Malawi	n/a	I. Kornfield	+	+
Astatotilapia calliptera	Salzburger et al. 2005	AY930090	Lake Malawi	n/a	I. Kornfield (A22)	+	+
Tropheus moorii	Salzburger et al. 2005	AY930091	Lake Tanganyika	n/a	E. Verheyen; 97	+	+
Cheilochromis euchilus	Salzburger et al. 2005	AY930092	Lake Malawi	n/a	I. Kornfield	+	+
Tropheus moorii	Salzburger et al. 2005	AY930093	Lake Tanganyika	n/a	E. Verheyen, 116		+
Pharyngochromis acuticeps	Salzburger et al. 2005	AY930094	Zambezi River, Zambia	n/a	C. Katongo / C. Sturmbauer		+
Thoracochromis brauschi	Salzburger et al. 2005	AY930095	Lake Fwa , Congo	n/a	Aquarium trade		+
Haplochromis sp. T13	Salzburger et al. 2005	AY930096	Upper Rusizi, Burundi	n/a	L. De Vos (T13-Aug 93)	+	+
Haplochromis obliquidens	Salzburger et al. 2005	AY930097	Lake Victoria	n/a	Aquarium trade	+	+
Sargochromis giardi	Salzburger et al. 2005	AY930098	Zambezi River, Zambia	n/a	C. Katongo / C. Sturmbauer		+
Cyclopharynx fwae	Salzburger et al. 2005	AY930099	Lake Fwa, Congo	n/a	U. Schliewen		+
Ctenochromis horei	Salzburger et al. 2005	AY930100	Lake Tanganyika	n/a	C. Sturmbauer/W. Salzburger		+
Haplochromis sp. 62	Salzburger et al. 2005	AY930101	Tanzania	n/a	L. De Vos (H62)	+	+
Haplochromis sp. 63	Salzburger et al. 2005	AY930102	Tanzania	n/a	L. De Vos (H63)	+	+
Haplochromis sp. 93/3	Salzburger et al. 2005	AY930103	Tanzania	n/a	L. Seegers (93/3)	+	+
Haplochromis sp. 93/40	Salzburger et al. 2005	AY930104	Tanzania	n/a	L. Seegers (93/40)	+	+
Haplochromis sp. 93/8	Salzburger et al. 2005	AY930105	Tanzania	n/a	L. Seegers (93/8)	+	+
Pseudocrenilabrus multicolor	Salzburger et al. 2005	AY930106	Tanzania	n/a	L. Seegers (91/137)		+
Haplochromis paludinosus	Salzburger et al. 2005	AY930107	Nanganga, Burundi	n/a	L. De Vos (T2-5/27/93)	+	+
Haplochromis gracilior	Salzburger et al. 2006	AY930079	Lake Kivu	n/a	E. Verheyen; K9	+	+
Congolapia bilineata	Schwarzer et al. 2011	JX157060	Itimbiri, DRC	n/a	ZSM		+
Lamprologus tigripictilis	Schwarzer et al. 2011	JX157061	Lower Congo, DRC	n/a	ZSM		+
Pseudocrenilabrus multicolor	Schwarzer et al. 2011	JX157062	Nile Delta, Egypt	n/a	ZSM	+	+
Orthochromis stormsi	Schwarzer et al. 2011	JX157063	Pool Malebo, DRC	n/a	ZSM		+
Orthochromis stormsi	Schwarzer et al. 2011	JX157064	Pool Malebo, DRC	n/a	ZSM		+
Orthochromis cf. stormsi 'Kisangani'	Schwarzer et al. 2011	JX157065	around Kisangani, DRC	n/a	ZSM		+
Orthochromis cf. stormsi 'Kisangani'	Schwarzer et al. 2011	JX157066	around Kisangani, DRC	n/a	ZSM		+
Orthochromis polyacanthus	Schwarzer et al. 2011	JX157067	LakeMweru, Zambia	n/a	EAWAG		+
Orthochromis aff. kalungwishiensis	Schwarzer et al. 2011	JX157068	LakeMweru, Zambia	n/a	EAWAG	+	+
Ctenochromis horei	Schwarzer et al. 2011	JX157069	Lake Tanganyika	n/a	CU		+
Ctenochromis horei	Schwarzer et al. 2011	JX157070	Lake Tanganyika	n/a	CU	+	+
Tropheus moorii	Schwarzer et al. 2011	JX157071	Lake Tanganyika	n/a	CU		+
Astatoreochromis alluaudi	Schwarzer et al. 2011	JX157072	Nile / Lake Victoria	n/a	EAWAG	+	+
Haplochromis burtoni	Schwarzer et al. 2011	JX157073	Lake Tanganyika	n/a	ZSM	+	+
Pseudotropheus socolofi	Schwarzer et al. 2011	JX157074	Lake Malawi	n/a	EAWAG	+	+

Labidochromis caeruleus	Schwarzer et al. 2011	JX157075	Lake Malawi	n/a	ZSM	+	+
Rhamphochromis sp.	Schwarzer et al. 2011	JX157076	Lake Malawi	n/a	ZSM	+	+
Sciaenochromis fryeri	Schwarzer et al. 2011	JX157077	Lake Malawi	n/a	ZSM	+	+
Astatotilapia desfontanii	Schwarzer et al. 2011	JX157078	Sahara, Tunesia	n/a	ZSM	+	+
Neochromis rufocaudalis	Schwarzer et al. 2011	JX157079	Nile / Lake Victoria	n/a	ZSM	+	+
Haplochromis sp. 'Kyoga'	Schwarzer et al. 2011	JX157080	Lake Kyoga, Uganda	n/a	ZSM	+	+
Haplochromis stappersii	Schwarzer et al. 2011	JX157081	Lake Tanganyika drainage, Burundi	n/a	ZSM	+	+
Haplochromis sp. 'Yaekama'	Schwarzer et al. 2011	JX157082	around Kisangani, DRC	n/a	ZSM	+	+
Haplochromis sp. 'Lake Rakai'	Schwarzer et al. 2011	JX157083	Nile / L. Rakai, Uganda	n/a	ZSM	+	+
Haplochromis sp. 'Lake Kijanebalola'	Schwarzer et al. 2011	JX157084	Nile / Lake Kijanebalola, Uganda	n/a	ZSM	+	+
Haplochromis thereuterion	Schwarzer et al. 2011	JX157085	Lake Victoria	n/a	ZSM	+	+
Haplochromis cf. polli 'Lefini'	Schwarzer et al. 2011	JX157086	Lefini River, ROC	n/a	MRAC		+
Haplochromis cf. polli 'Lefini'	Schwarzer et al. 2011	JX157087	Lefini River, ROC	n/a	MRAC		+
Haplochromis polli	Schwarzer et al. 2011	JX157088	Lower Congo River	n/a	ZSM		+
Haplochromis polli	Schwarzer et al. 2011	JX157089	Lower Congo River	n/a	ZSM		+
Haplochromis oligacanthus	Schwarzer et al. 2011	JX157090	Ubangi River, CAR	n/a	ZSM		+
Haplochromis oligacanthus	Schwarzer et al. 2011	JX157091	Ubangi River, CAR	n/a	ZSM		+
Haplochromis fasciatus	Schwarzer et al. 2011	JX157092	Lower Congo River	n/a	ZSM		+
Haplochromis fasciatus	Schwarzer et al. 2011	JX157093	Lower Congo River	n/a	ZSM	+	+
Haplochromis demeusii	Schwarzer et al. 2011	JX157094	Lower Congo River	n/a	ZSM		+
Haplochromis demeusii	Schwarzer et al. 2011	JX157095	Lower Congo River	n/a	ZSM	+	+
Haplochromis sp. 'Sanzikwa'	Schwarzer et al. 2011	JX157096	Sanzikwa River, DRC	n/a	ZSM		+
Haplochromis sp. 'Sanzikwa'	Schwarzer et al. 2011	JX157097	Sanzikwa River, DRC	n/a	ZSM	+	+
Haplochromis cf. bakongo	Schwarzer et al. 2011	JX157098	Kwilu River, DRC	n/a	ZSM		+
Haplochromis cf. bakongo	Schwarzer et al. 2011	JX157099	Kwilu River, DRC	n/a	ZSM		+
Haplochromis snoeksi	Schwarzer et al. 2011	JX157100	Inkisi River, DRC	n/a	MRAC	+	+
Thoracochromis callichromus	Schwarzer et al. 2011	JX157101	Lake Fwa, DRC	n/a	AMNH		+
Thoracochromis callichromus	Schwarzer et al. 2011	JX157102	Lake Fwa, DRC	n/a	AMNH		+
Cyclopharynx schwetzi	Schwarzer et al. 2011	JX157103	Lake Fwa, DRC	n/a	AMNH		+
Thoracochromis brauschi	Schwarzer et al. 2011	JX157104	Lake Fwa, DRC	n/a	AMNH		+
Schwetzochromis neodon	Schwarzer et al. 2011	JX157105	Lake Fwa, DRC	n/a	AMNH		+
Haplochromis stigmatogenys	Schwarzer et al. 2011	JX157106	Kasai River, DRC	n/a	AMNH		+
Haplochromis stigmatogenys	Schwarzer et al. 2011	JX157107	Kasai River, DRC	n/a	AMNH		+
Haplochromis sp. 'Kwango'	Schwarzer et al. 2011	JX157108	Kwango River, DRC	n/a	ZSM		+
Haplochromis sp. 'Kwango'	Schwarzer et al. 2011	JX157109	Kwango River, DRC	n/a	ZSM		+
Orthochromis torrenticola	Schwarzer et al. 2011	JX157110	Lufira, DRC	n/a	ZSM		+
Orthochromis torrenticola	Schwarzer et al. 2011	JX157111	Lufira, DRC	n/a	ZSM		+
Pharyngochromis sp. 'yellow lip'	Schwarzer et al. 2011	JX157112	Kwanza / Middel Kwanza (Angola)	n/a	SAIAB		+
Pharyngochromis sp. 'yellow lip'	Schwarzer et al. 2011	JX157113	Kwanza / Middel Kwanza, Angola	n/a	SAIAB		+
Pharyngochromis sp. 'yellow lip'	Schwarzer et al. 2011	JX157114	Kwanza / Middel Kwanza, Angola	n/a	SAIAB		+
Pharyngochromis sp. 'white tip'	Schwarzer et al. 2011	JX157115	Kwanza / Upper Lucalla, Angola	n/a	SAIAB		+
Pharyngochromis sp. 'white tip'	Schwarzer et al. 2011	JX157116	Kwanza / Upper Lucalla (Angola)	n/a	SAIAB		+
Serranochromis sp. 'red scales'	Schwarzer et al. 2011	JX157117	Kwanza / Upper Lucalla, Angola	n/a	SAIAB		+
Serranochromis sp. 'red scales'	Schwarzer et al. 2011	JX157118	Kwanza / Upper Lucalla, Angola	n/a	SAIAB		+
Pharyngochromis sp. 'yellow fins'	Schwarzer et al. 2011	JX157119	Kwanza / Upper Kwanza, Angola	n/a	SAIAB		+
Serranochromis sp. 'yellow fins'	Schwarzer et al. 2011	JX157120	Kwanza / Upper Kwanza, Angola	n/a	SAIAB	+	+
Serranochromis sp. 'black and white'	Schwarzer et al. 2011	JX157121	Kwanza / Upper Kwanza, Angola	n/a	SAIAB		+
Pharyngochromis acuticeps	Schwarzer et al. 2011	JX157122	Zambezi, Namibia	n/a	ZSM		+
Serranochromis robustus	Schwarzer et al. 2011	JX157123	Zambezi, Namibia	n/a	ZSM		+
Serranochromis macrocephalus	Schwarzer et al. 2011	JX157124	Zambezi, Namibia	n/a	ZSM		+
Serranochromis angusticeps	Schwarzer et al. 2011	JX157125	Zambezi, Namibia	n/a	ZSM		+
Serranochromis altus	Schwarzer et al. 2011	JX157126	Zambezi, Namibia	n/a	ZSM		+
Haplochromis elegans	Wagner et al. 2012	JQ950379	n/a	n/a	EAWAG, KAT_10	+	
Astatotilapia flavijosephi	Wagner et al. 2012	JQ950380	n/a	n/a	EAWAG, voucher 14	+	
Haplochromis tweddlei	Wagner et al. 2012	JQ950384	n/a	n/a	EAWAG, voucher 2_B6	+	
Haplochromis paludinosus	Weiss et al. unpublished	KJ176274	n/a	n/a	ZSM, P-AA-0595	+	
