# Supplementary material for: Back to Tanganyika: a case of recent trans-species-flock dispersal in East African haplochromine cichlid fishes
Source: R Soc Open Sci. 2015 Mar 4;2(3):140498. doi: 10.1098/rsos.140498 (PMC4448823; doi:10.1098/rsos.140498)
Supplement: Table_S3_acc.pdf [file rsos140498supp3.pdf]

Supplementary table 3: List of the 182 haplochromine specimens and their mitochondrial control region (d-loop) accession numbers. Specified are the original publications and their sample information including haplotype number following Verheyen et al. 2003 and this study. Haplotypes used in figure 1(d) are indicated with an asterisk.

Species	Published in	Accession number	Locality	Collected by	SampleID	Haplotype in Verheyen et al. 2003 / this study
Haplochromis simpsoni	Nagl et al. 2000	AF213518	Lake Nabugabo	-	Gasi589	77
Haplochromis beadlei	Nagl et al. 2000	AF213519	Lake Nabugabo	-	Pabe593	77
Haplochromis laparogramma	Nagl et al. 2000	AF213520	Lake Victoria	-	Yila179	89
Haplochromis laparogramma	Nagl et al. 2000	AF213521	Lake Victoria	-	Yila335	80
Haplochromis laparogramma	Nagl et al. 2000	AF213522	Rusinga / Lake Victoria	-	Yila6937	25
Haplochromis lividus	Nagl et al. 2000	AF213523	Lake Victoria	-	Hali327	93
Haplochromis nubila	Nagl et al. 2000	AF213524	Lakes Nabugabo, Kayina and Kayania	-	Asnu	92*
Haplochromis chilotes	Nagl et al. 2000	AF213525	Rusinga / Lake Victoria	-	Pach	98
Haplochromis cinctus	Nagl et al. 2000	AF213526	Lake Victoria	-	Enci	77*
Haplochromis melanopterus	Nagl et al. 2000	AF213527	Lake Victoria	-	Lime	95
Neochromis nigricans	Nagl et al. 2000	AF213528	Lake Victoria	-	Neni	121
Haplochromis plagiodon	Nagl et al. 2000	AF213529	Lake Victoria	-	Papl	105
Haplochromis riponianus	Nagl et al. 2000	AF213530	Lake Victoria	-	Psri	102
Haplochromis fischeri	Nagl et al. 2000	AF213531	Lake Victoria	-	Ptsa	122
Haplochromis xenognathus	Nagl et al. 2000	AF213532	Anyanga / Lake Victoria	-	Ptxe6864	113
Haplochromis xenognathus	Nagl et al. 2000	AF213533	Anyanga / Lake Victoria	-	Ptxe6865	110
Haplochromis xenognathus	Nagl et al. 2000	AF213534	Mwanza Gulf / Lake Victoria	-	Ptxe326	109
Haplochromis xenognatus	Nagl et al. 2000	AF213535	Lake Victoria	-	Ptxe350	118*
Haplochromis nubilus	Nagl et al. 2000	AF213536	Lake Victoria	-	Asnu586	117
Prognathochromis venator	Nagl et al. 2000	AF213537	Lakes Nabugabo, Kayina and Kayania	-	Prve687	81
Prognathochromis venator	Nagl et al. 2000	AF213538	Lakes Nabugabo, Kayina and Kayania	-	Prve691	81
Haplochromis chilotes	Nagl et al. 2000	AF213539	Anyanga / Lake Victoria	-	Pach5721	79
Haplochromis chilotes	Nagl et al. 2000	AF213540	Lake Victoria	-	Pach5722	90
Haplochromis sp.'rockkribensis'	Nagl et al. 2000	AF213541	Lake Victoria	-	Haro486	108
Haplochromis sp.'rockkribensis'	Nagl et al. 2000	AF213542	Muhuru / Lake Victoria	-	Haro6745	75
Haplochromis sp.'velvetblack'	Nagl et al. 2000	AF213543	Lake Victoria	-	Havb21	115
Neochromis nigricans	Nagl et al. 2000	AF213544	Lake Victoria	-	Neni309	99
Neochromis nigricans	Nagl et al. 2000	AF213545	Lake Victoria	-	Neni817	96
Haplochromis plagiodon	Nagl et al. 2000	AF213546	Lake Victoria	-	Papl73	104
Haplochromis plagiodon	Nagl et al. 2000	AF213547	Lake Victoria	-	Papl160	91
Haplochromis plagiodon	Nagl et al. 2000	AF213548	Lake Victoria	-	Papl201	92
Haplochromis fischeri	Nagl et al. 2000	AF213549	Lake Victoria	-	Ptsa320	106
Haplochromis velifer	Nagl et al. 2000	AF213550	Lakes Nabugabo, Kayina and Kayania	-	Asve616	88
Haplochromis velifer	Nagl et al. 2000	AF213551	Lakes Nabugabo, Kayina and Kayania	-	Asve605	94
Haplochromis velifer	Nagl et al. 2000	AF213552	Lakes Nabugabo, Kayina and Kayania	-	Asve619	114
Haplochromis velifer	Nagl et al. 2000	AF213553	Lakes Nabugabo, Kayina and Kayania	-	Asve663	107
Haplochromis sp.'rockkribensis'	Nagl et al. 2000	AF213554	Lake Victoria	-	Haro6747	76*
Haplochromis sp.	Nagl et al. 2000	AF213555	WogoRiver / LakeRukwa	-	1514	27*
Haplochromis sp.	Nagl et al. 2000	AF213556	MyungaRiver / LakeRukwa	-	1605	28*
Haplochromis sp.	Nagl et al. 2000	AF213557	Kasenyi / Lake George	-	8831	73*
Haplochromis sp.	Nagl et al. 2000	AF213558	Kasenyi / Lake George	-	HT-8833	68
Haplochromis sp.	Nagl et al. 2000	AF213559	Kasenyi / Lake George	-	HT-87868786	5
Haplochromis sp.	Nagl et al. 2000	AF213560	Kasenyi / Lake George	-	HT-8801	64
Haplochromis sp.	Nagl et al. 2000	AF213561	Kasenyi / Lake George	-	HT-8837	1
Haplochromis sp.	Nagl et al. 2000	AF213562	Kasenyi / Lake George	-	HT-88348834	41
Haplochromis sp.	Nagl et al. 2000	AF213563	Kashaka / Lake George	-	HT-8924	43
Haplochromis sp.	Nagl et al. 2000	AF213564	Katwe / LakeEdward	-	HT-8880	26
Haplochromis sp.	Nagl et al. 2000	AF213566	Katwe / LakeEdward	-	HT-8879	71
Haplochromis sp.	Nagl et al. 2000	AF213567	Katwe / LakeEdward	-	HT-87688768	40
Haplochromis sp.	Nagl et al. 2000	AF213568	Katwe / LakeEdward	-	HT-8773	45
Haplochromis sp.	Nagl et al. 2000	AF213569	Katwe / LakeEdward	-	8777	46*
Haplochromis sp.	Nagl et al. 2000	AF213570	Katwe / LakeEdward	-	HT-8778	2
Haplochromis sp.	Nagl et al. 2000	AF213571	Bugoigo / LakeAlbert	-	HT-9049	66
Haplochromis sp.	Nagl et al. 2000	AF213572	Butiaba / LakeAlbert	-	HT-8990	69
Haplochromis sp.	Nagl et al. 2000	AF213573	Butiaba / LakeAlbert	-	HT-9003	44
Haplochromis sp.	Nagl et al. 2000	AF213574	Butiaba / LakeAlbert	-	HT-9019	42
Haplochromis sp.	Nagl et al. 2000	AF213575	LakeLutoto / Uganda	-	HT-8692	30
Haplochromis sp.	Nagl et al. 2000	AF213576	LakeLutoto / Uganda	-	HT-8694	31
Haplochromis sp.	Nagl et al. 2000	AF213577	LakeLutoto / Uganda	-	HT-8687	32
Haplochromis sp.	Nagl et al. 2000	AF213578	LakeChibwera / Uganda	-	HT-8947	62
Haplochromis sp.	Nagl et al. 2000	AF213579	LakeChibwera / Uganda	-	HT-8950	60
Haplochromis sp.	Nagl et al. 2000	AF213580	LakeChibwera / Uganda	-	HT-8948	61
Haplochromis sp.	Nagl et al. 2000	AF213581	LakeWamala / Lake VictoriaRegion	-	HT-8632	111

Haplochromis sp.	Nagl et al. 2000	AF213582	KatongaRiver / Lake VictoriaRegion	-	HT-8678	116
Haplochromis sp.	Nagl et al. 2000	AF213583	KatongaRiver / Lake VictoriaRegion	-	HT-8680	112
Haplochromis sp.	Nagl et al. 2000	AF213584	Kazinga Channel / L.Edward and George	-	HT-8741	70
Haplochromis sp.	Nagl et al. 2000	AF213585	Kazinga Channel / L.Edward and George	-	HT-8711	4
Haplochromis sp.	Nagl et al. 2000	AF213586	Kazinga Channel / L.Edward and George	-	HT-8718	3
Haplochromis sp.	Nagl et al. 2000	AF213587	Kazinga Channel / L.Edward and George	-	HT-87228722	39
Haplochromis sp.	Nagl et al. 2000	AF213588	MigoriRiver / Lake Victoria	-	HT-6701	87
Haplochromis sp.	Nagl et al. 2000	AF213589	Malagarazi River	-	HT-1006	na
Haplochromis sp.	Nagl et al. 2000	AF213590	Malagarazi River	-	HT-1011	na
Haplochromis sp.	Nagl et al. 2000	AF213591	Malagarazi River	-	HT-1510	na
Haplochromis sp.	Nagl et al. 2000	AF213592	Malagarazi River	-	HT-1531	na
Haplochromis sp.	Nagl et al. 2000	AF213593	Malagarazi River	-	HT-1590	na
Haplochromis sp.	Nagl et al. 2000	AF213594	Malagarazi River	-	HT-1591	na
Haplochromis sp.	Nagl et al. 2000	AF213595	Lupa River	-	HT-1597	na
Haplochromis sp.	Nagl et al. 2000	AF213596	Piti River	-	HT-1598	na
Haplochromis sp.	Nagl et al. 2000	AF213597	Piti River	-	HT-1546	na
Haplochromis sp.	Nagl et al. 2000	AF213598	Piti River	-	HT-1547	na
Haplochromis sp.	Nagl et al. 2000	AF213599	Pangani River	-	HT-1076	na
Haplochromis sp.	Nagl et al. 2000	AF213600	Pangani River	-	HT-1501	na
Haplochromis sp.	Nagl et al. 2000	AF213601	Wogo River / Lake Rukwa	-	HT-1636	na
Haplochromis sp.	Nagl et al. 2000	AF213602	Wogo River / Lake Rukwa	-	HT-1635	na
Haplochromis sp.	Nagl et al. 2000	AF213603	Wogo River / Lake Rukwa	-	HT-1515	na
Haplochromis sp.	Negletal.2002	AF213604	Pangani River	-	HT-1530	na
Haplochromis sp.	Negletal.2001	AF213605	Lake Chala	-	HT-1738	na
Haplochromis sp.	Negletal.2003	AF213606	Lake Babati	-	HT-6249	na
Haplochromis sp.	Nagl et al. 2000	AF213607	Lake Manyara	-	HT-1537	na
Haplochromis sp.	Nagl et al. 2000	AF213608	Malagarazi River	-	HT-1601	na
Haplochromis sp.	Nagl et al. 2000	AF213609	Kazinga Channel / L. Edwardand George	-	HT-8746	na
Haplochromis sp.	Nagl et al. 2000	AF213610	Lake George	-	HT-8785	na
Haplochromis sp.	Nagl et al. 2000	AF213611	Lake George	-	HT-8903	na
Haplochromis sp.	Nagl et al. 2000	AF213612	Lake George	-	HT-8911	na
Haplochromis sp.	Nagl et al. 2000	AF213613	Malagarazi River	-	HT-1533	na
Haplochromis sp.	Nagl et al. 2000	AF213614	Malagarazi River	-	HT-1609	na
Astatoreochromis alluaudi	Nagl et al. 2000	AF213616	Lake Victoria	-	Asal6744	na
Astatoreochromis alluaudi	Nagl et al. 2000	AF213617	Lake Victoria	-	Asal5928	na
Pseudotropheus sp.'msobo'	Nagl et al. 2000	AF213622	Lake Malawi	-	Psms5170	na
Labeotropheus trewavasae	Nagl et al. 2000	AF213623	Lake Malawi	-	Latr5493	na
Haplochromis burtoni	Stiassny et al. 1994	AF400710	-	-	8153	na
Limnochromis auritus	Sturmbauer & Meyer 1992	AF400728	Lake Tanganyika	-	27749	na
Petrochromis orthognathus	Stiassny et al. 1994	AF400734	Lake Tanganyika	-	28818	na
Haplochromis astatodon	Verheyen et al. 2003	AY226611	Lake Kivu	E.Verheyen	K114	7
Haplochromis astatodon	Verheyen et al. 2003	AY226611	Lake Kivu	E.Verheyen	K114	7*
Haplochromis insidiae	Verheyen et al. 2003	AY226627	Lake Kivu	E.Verheyen	K080	8
Haplochromis sp.nigroides / scheffersi	Verheyen et al. 2003	AY226629	Lake Kivu	E.Verheyen	K146	9
Haplochromis astatodon	Verheyen et al. 2003	AY226631	Lake Kivu	E.Verheyen	K119	10
Haplochromis astatodon	Verheyen et al. 2003	AY226632	Lake Kivu	E.Verheyen	K131	11
Haplochromis paucidens	Verheyen et al. 2003	AY226633	Lake Kivu	E.Verheyen	K112	12
Haplochromis paucidens	Verheyen et al. 2003	AY226640	Lake Kivu	E.Verheyen	K022	13
Haplochromis paucidens	Verheyen et al. 2003	AY226641	Lake Kivu	E.Verheyen	K034	14
Haplochromis sp.crebridens / olivaceus	Verheyen et al. 2003	AY226642	Lake Kivu	E.Verheyen	K036	15
Haplochromis astatodon	Verheyen et al. 2003	AY226643	Lake Kivu	E.Verheyen	K127	16
Haplochromis sp.crebridens / olivaceus	Verheyen et al. 2003	AY226646	Lake Kivu	E.Verheyen	K060	17
Haplochromis scheffersi	Verheyen et al. 2003	AY226647	Lake Kivu	E.Verheyen	K111	18
Haplochromis graueri	Verheyen et al. 2003	AY226648	Lake Kivu	E.Verheyen	K118	19
Haplochromis graueri	Verheyen et al. 2003	AY226649	Lake Kivu	E.Verheyen	K012	20
Haplochromis astatodon	Verheyen et al. 2003	AY226650	Lake Kivu	E.Verheyen	K115	21
Haplochromis astatodon	Verheyen et al. 2003	AY226651	Lake Kivu	E.Verheyen	K124	22
Haplochromis astatodon	Verheyen et al. 2003	AY226652	Lake Kivu	E.Verheyen	K076	23
Haplochromis astatodon	Verheyen et al. 2003	AY226654	Lake Kivu	E.Verheyen	K132	24
Haplochromis astatodon	Verheyen et al. 2003	AY226655	Lake Kivu	E.Verheyen	K51	25*
Haplochromis occultidens	Verheyen et al. 2003	AY226666	Lake Kivu	E.Verheyen	K030	33
Haplochromis graueri	Verheyen et al. 2003	AY226668	Lake Kivu	E.Verheyen	K001	36
Haplochromis astatodon	Verheyen et al. 2003	AY226669	Lake Kivu	E.Verheyen	K116	37
Haplochromis sp.crebridens / olivaceus	Verheyen et al. 2003	AY226670	Lake Kivu	E.Verheyen	K057	38
Haplochromis astatodon	Verheyen et al. 2003	AY226671	Lake Kivu	E.Verheyen	K135	47*
Haplochromis paucidens	Verheyen et al. 2003	AY226687	Lake Kivu	E.Verheyen	K056	48

Haplochromis nigroides	Verheyen et al. 2003	AY226688	Lake Kivu	E.Verheyen	K028	49
Haplochromis astatodon	Verheyen et al. 2003	AY226691	Lake Kivu	E.Verheyen	K152	50
Haplochromis astatodon	Verheyen et al. 2003	AY226692	Lake Kivu	E.Verheyen	K138	51
Haplochromis paucidens	Verheyen et al. 2003	AY226694	Lake Kivu	E.Verheyen	K058	53
Haplochromis microchrysomelas	Verheyen et al. 2003	AY226695	Lake Kivu	E.Verheyen	K113	54
Haplochromis astatodon	Verheyen et al. 2003	AY226697	Lake Kivu	E.Verheyen	K120	55
Haplochromis microchrysomelas	Verheyen et al. 2003	AY226699	Lake Kivu	E.Verheyen	K142	56*
Haplochromis paucidens	Verheyen et al. 2003	AY226712	Lake Kivu	E.Verheyen	K174	57
Haplochromis crebridens	Verheyen et al. 2003	AY226714	Lake Kivu	E.Verheyen	K177	58
Haplochromis adolfifrederici	Verheyen et al. 2003	AY226715	Lake Kivu	E.Verheyen	K169	59
Haplochromis crebridens	Verheyen et al. 2003	AY226716	Lake Kivu	E.Verheyen	K063	74
Haplochromis sp.	Verheyen et al. 2003	AY226719	Cohoha / Bugesera Lakes	J.Snoeks	D9	82*
Haplochromis sp.	Verheyen et al. 2003	AY226720	Cohoha / Bugesera Lakes	J.Snoeks	B4	83
Haplochromis sp.	Nagl et al. 2000	AY226723	Rweru / Bugesera Lakes	-	R1	84
Haplochromis sp.	Verheyen et al. 2003	AY226726	Cohoha / Bugesera Lakes	J.Snoeks	D8	85
Haplochromis sp.	Verheyen et al. 2003	AY226727	Kachera / Uganda	E.Schraml	9803	6
Haplochromis sp.	Verheyen et al. 2003	AY226734	Victoria Nile	E.Schraml	9791	29
Haplochromis sp.	Verheyen et al. 2003	AY226735	Mugogo / Uganda	E.Schraml	9784	32
Haplochromis sp.	Verheyen et al. 2003	AY226736	aquarium trade	E.Schraml	9808	63
Haplochromis squamipinnis	Verheyen et al. 2003	AY226747	Lake Edward	E.Schraml	9813	65
Haplochromis sp.	Verheyen et al. 2003	AY226752	Nyamusingire / Uganda	E.Schraml	9765	67
Haplochromis sp.	Verheyen et al. 2003	AY226758	Nakivali / Uganda	E.Schraml	9721	72
Haplochromis sp.	Verheyen et al. 2003	AY226759	Lake Victoria	E.Schraml	9707	77
Haplochromis sp.	Verheyen et al. 2003	AY226761	Nawampasa / Lake Kyoga	E.Schraml	9788	78
Haplochromis sp.	Verheyen et al. 2003	AY226762	Lake Victoria	E.Schraml	9801	86
Haplochromis sp.	Verheyen et al. 2003	AY226763	Lake Victoria	E.Schraml	9713	91*
Haplochromis sp.	Verheyen et al. 2003	AY226764	Lake Victoria	E.Schraml	9706	92
Haplochromis sp.	Verheyen et al. 2003	AY226765	Lake Victoria	E.Schraml	9715	92
Haplochromis sp.	Verheyen et al. 2003	AY226766	Nawamapasa / Lake Kyoga	E.Schraml	9789	97*
Haplochromis sp.	Verheyen et al. 2003	AY226767	Lake Victoria	E.Schraml	9812	100
Haplochromis sp.	Verheyen et al. 2003	AY226768	Mulehe / Kabale Lakes	E.Schraml	9764	101*
Haplochromis sp.	Verheyen et al. 2003	AY226769	Lake Victoria	E.Schraml	9704	101
Haplochromis sp.	Verheyen et al. 2003	AY226779	Lake Victoria	E.Schraml	9703	103
Haplochromis sp.	Verheyen et al. 2003	AY226781	Bunyoni / Kabale Lakes	-	9727	119
Haplochromis sp.	Verheyen et al. 2003	AY226784	Bunyoni / Kabale Lakes	E.Schraml	9741	120
Haplochromis burtoni	Verheyen et al. 2003	AY226785	Cohoha / Bugesera Lakes	J.Snoeks	B6	na
Astatoreochromis alluaudi	Verheyen et al. 2003	AY226787	Cohoha / Bugesera Lakes	J.Snoeks	E9	na
Haplochromis gracilior	Verheyen et al. 2003	AY226788	Lake Kivu	-	K008	na
Haplochromis gracilior	Verheyen et al. 2003	AY226789	Lake Kivu	-	K009	na
Haplochromis gracilior	Verheyen et al. 2003	AY226790	Lake Kivu	-	K010	na
Thoracochromis brauschi	Verheyen et al. 2003	AY226791	Lac Fwa	Paul	9792	na
Serranochromis sp.WWS-2003	Verheyen et al. 2003	AY226792	Lake Mweru-Wantipa	T.Reuter	9793	na
Haplochromis stappersii	Salzburgeretal.2005	AY929941	Malagarazi River	L.DeVos	5-6 / 25 / 92	M3*
Haplochromis sp.	Salzburgeretal.2005	AY929992	Tanzania	L.Seegers	93 / 8	LR2*
Haplochromis sp.	Salzburgeretal.2005	AY930015	Tanzania	L.Seegers	92 / 12	LR1*
Cyrtocara moorii	Sturmbauer & Meyer 1992	U12554	Lake Tanganyika	-	30882	na
Haplochromis stappersii "Malagarasi1"	this study	KJ955382	Malagarazi River / Burundi	G.Banyankimbona	MRAC1840	M1*
Haplochromis stappersii "Malagarasi2"	this study	KJ955384	Malagarazi River / Burundi	G.Banyankimbona	MRAC1847	M1*
Haplochromis stappersii "Malagarasi3"	this study	KJ955385	Malagarazi River / Burundi	G.Banyankimbona	MRAC12034	M1*
Haplochromis stappersii "Malagarasi4"	this study	KJ955383	Malagarazi River / Burundi	G.Banyankimbona	MRAC12087	M2*
Haplochromis sp. "Chipwa"	this study	KJ955386	Kalambo River / Zambia	W.Salzburger	CH4	HLT*
Haplochromis sp. "Chipwa"	this study	KJ955387	Lufubu River / Zambia	W.Salzburger	LU2	HLT*
Haplochromis stappersii "Rusizi"	this study	KJ955381	Gatumbamarsh, Rusizi River	G.Banyankimbona	MRAC6334	RR*
