# Supplementary material for: Back to Tanganyika: a case of recent trans-species-flock dispersal in East African haplochromine cichlid fishes
Source: R Soc Open Sci. 2015 Mar 4;2(3):140498. doi: 10.1098/rsos.140498 (PMC4448823; doi:10.1098/rsos.140498)
Supplement: S1.pdf [file rsos140498supp4.pdf]

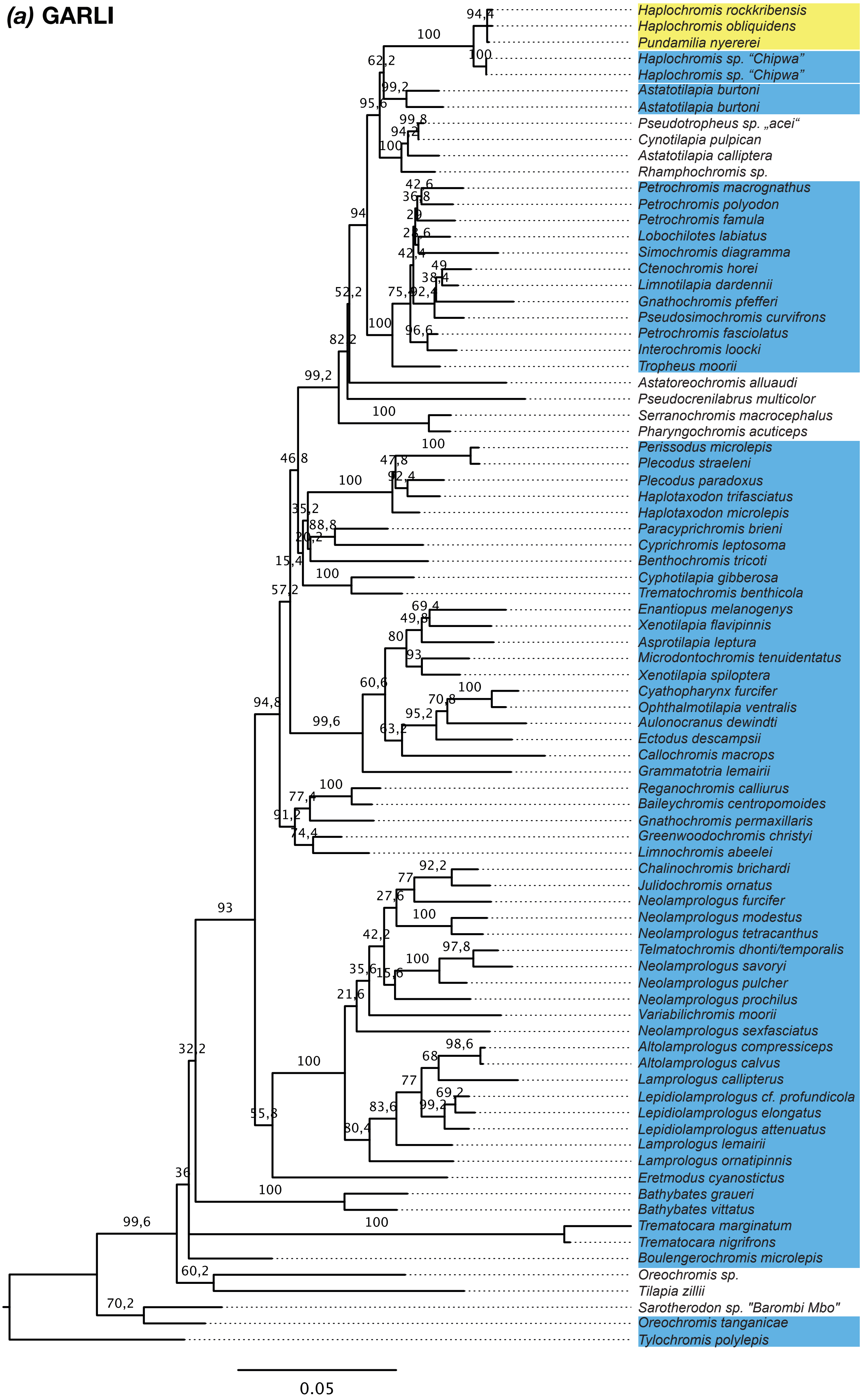

Maximum likelihood (*a*) and Bayesian (*b*) tree based on the concatenated dataset (table S1). All bootstrap support values and posterior probabilities are plotted. The geographical origin of the specimen is indicated in color (blue = Lake Tanganyika; yellow = Lake Victoria; other locations are not further indicated).

(b) MrBayes

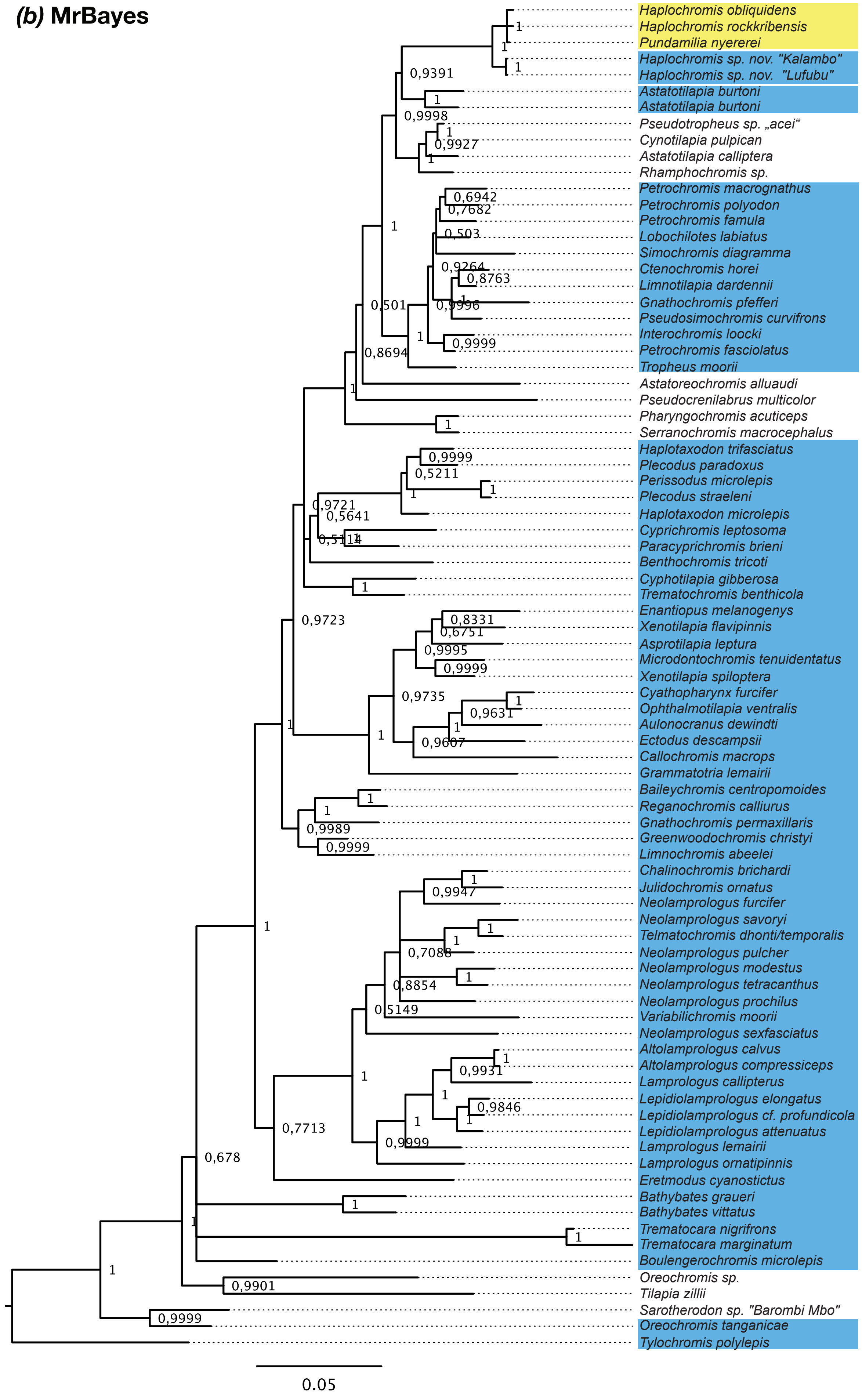

Haplochromis obliquidens
Haplochromis rockkribensis
Pundamilia nyererei
Haplochromis sp. nov. "Kalambo"
Haplochromis sp. nov. "Lufubu"
Astatotilapia burtoni
Astatotilapia burtoni
Pseudotropheus sp. „acei“
Cynotilapia pulpican
Astatotilapia calliptera
Rhamphochromis sp.
Petrochromis macrognathus
Petrochromis polyodon
Petrochromis famula
Lobochilotes labiatus
Simochromis diagramma
Ctenochromis horei
Limnotilapia dardennii
Gnathochromis pfefferi
Pseudosimochromis curvifrons
Interochromis loocki
Petrochromis fasciolatus
Tropheus moorii
Astatoreochromis alluaudi
Pseudocrenilabrus multicolor
Pharyngochromis acuticeps
Serranochromis macrocephalus
Haplotaxodon trifasciatus
Plecodus paradoxus
Perissodus microlepis
Plecodus straeleni
Haplotaxodon microlepis
Cyprichromis leptosoma
Paracyprichromis brieni
Benthochromis tricoti
Cyphotilapia gibberosa
Trematochromis benthicola
Enantiopus melanogenys
Xenotilapia flavipinnis
Asprotilapia leptura
Microdontochromis tenuidentatus
Xenotilapia spiloptera
Cyathopharynx furcifer
Ophthalmotilapia ventralis
Aulonocranus dewindti
Ectodus descampsii
Callochromis macrops
Grammatotria lemairii
Baileychromis centropomoides
Reganochromis calliurus
Gnathochromis permaxillaris
Greenwoodochromis christyi
Limnochromis abeelei
Chalinochromis brichardi
Julidochromis ornatus
Neolamprologus furcifer
Neolamprologus savoryi
Telmatochromis dhonti/temporalis
Neolamprologus pulcher
Neolamprologus modestus
Neolamprologus tetracanthus
Neolamprologus prochilus
Variabilichromis moorii
Neolamprologus sexfasciatus
Altolamprologus calvus
Altolamprologus compressiceps
Lamprologus callipterus
Lepidiolamprologus elongatus
Lepidiolamprologus cf. profundicola
Lepidiolamprologus attenuatus
Lamprologus lemairii
Lamprologus ornatipinnis
Eretmodus cyanostictus
Bathybates graueri
Bathybates vittatus
Trematocara nigrifrons
Trematocara marginatum
Boulengerochromis microlepis
Oreochromis sp.
Tilapia zillii
Sarotherodon sp. "Barombi Mbo"
Oreochromis tanganicae
Tylochromis polylepis
0,9391
0,9998
0,9927
0,6942
0,7682
0,503
0,9264
0,8763
0,9996
0,501
0,9999
0,8694
0,9999
0,5211
0,9721
0,5641
0,5114
0,9723
0,8331
0,6751
0,9995
0,9999
0,9735
0,9631
0,9607
0,9989
0,9999
0,9947
0,7088
0,8854
0,5149
0,9931
0,9846
0,9999
0,7713
0,678
0,9901
0,9999
0.05
