# Supplementary material for: Back to Tanganyika: a case of recent trans-species-flock dispersal in East African haplochromine cichlid fishes
Source: R Soc Open Sci. 2015 Mar 4;2(3):140498. doi: 10.1098/rsos.140498 (PMC4448823; doi:10.1098/rsos.140498)
Supplement: S2.pdf [file rsos140498supp5.pdf]

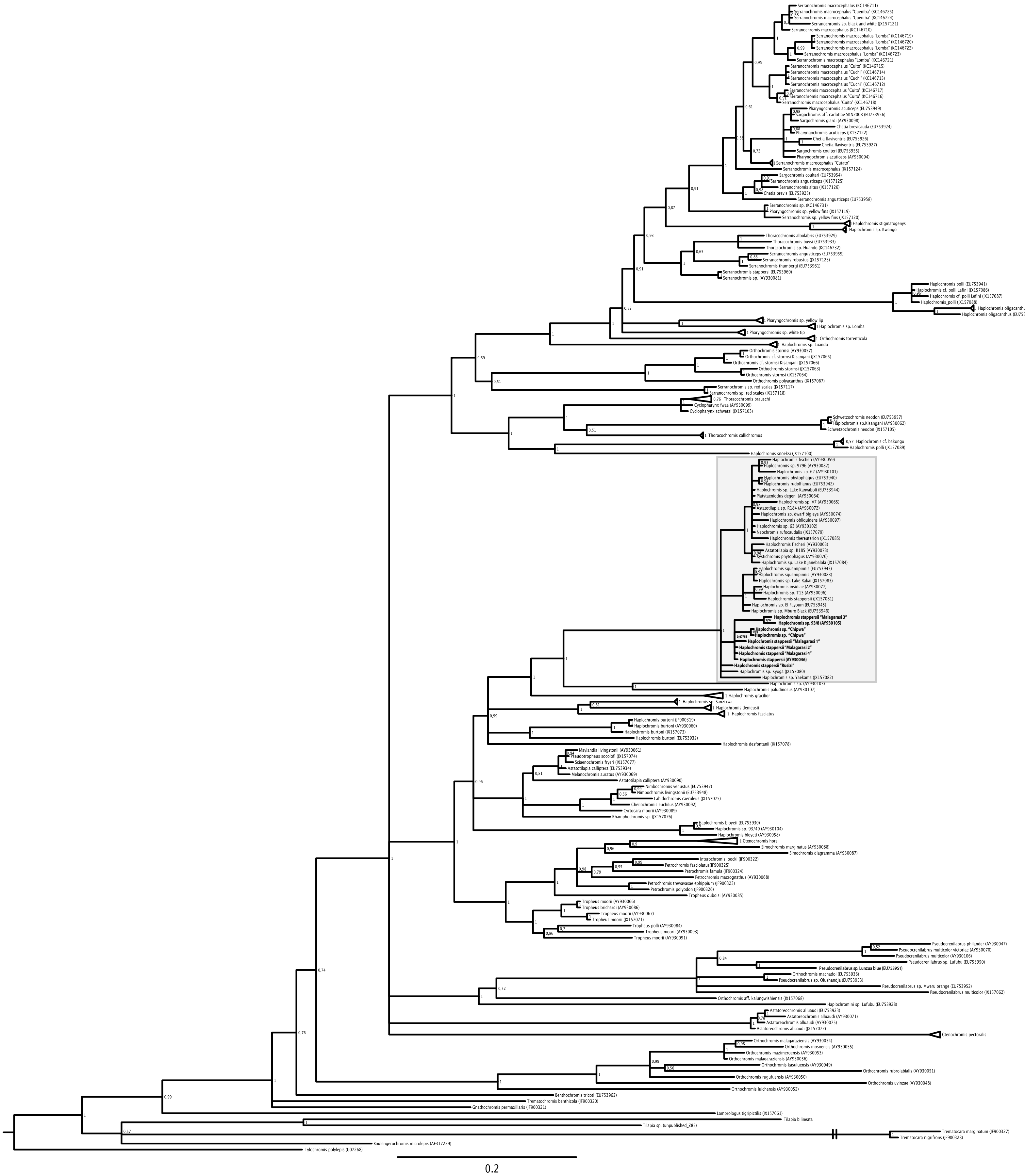

Supplementary figure 2: MrBayes 50% majority rule consensus tree with branch lengths based on the ND2 data set (table S2). Posterior probabilities ≥ 0.5 are plotted. The grey box represents the Lake Victoria Region superflock.
