# Supplementary figures and images for: Back to Tanganyika: a case of recent trans-species-flock dispersal in East African haplochromine cichlid fishes
Source: R Soc Open Sci. 2015 Mar 4;2(3):140498. doi: 10.1098/rsos.140498 (PMC4448823; doi:10.1098/rsos.140498)

Lake Victoria Region Superflock

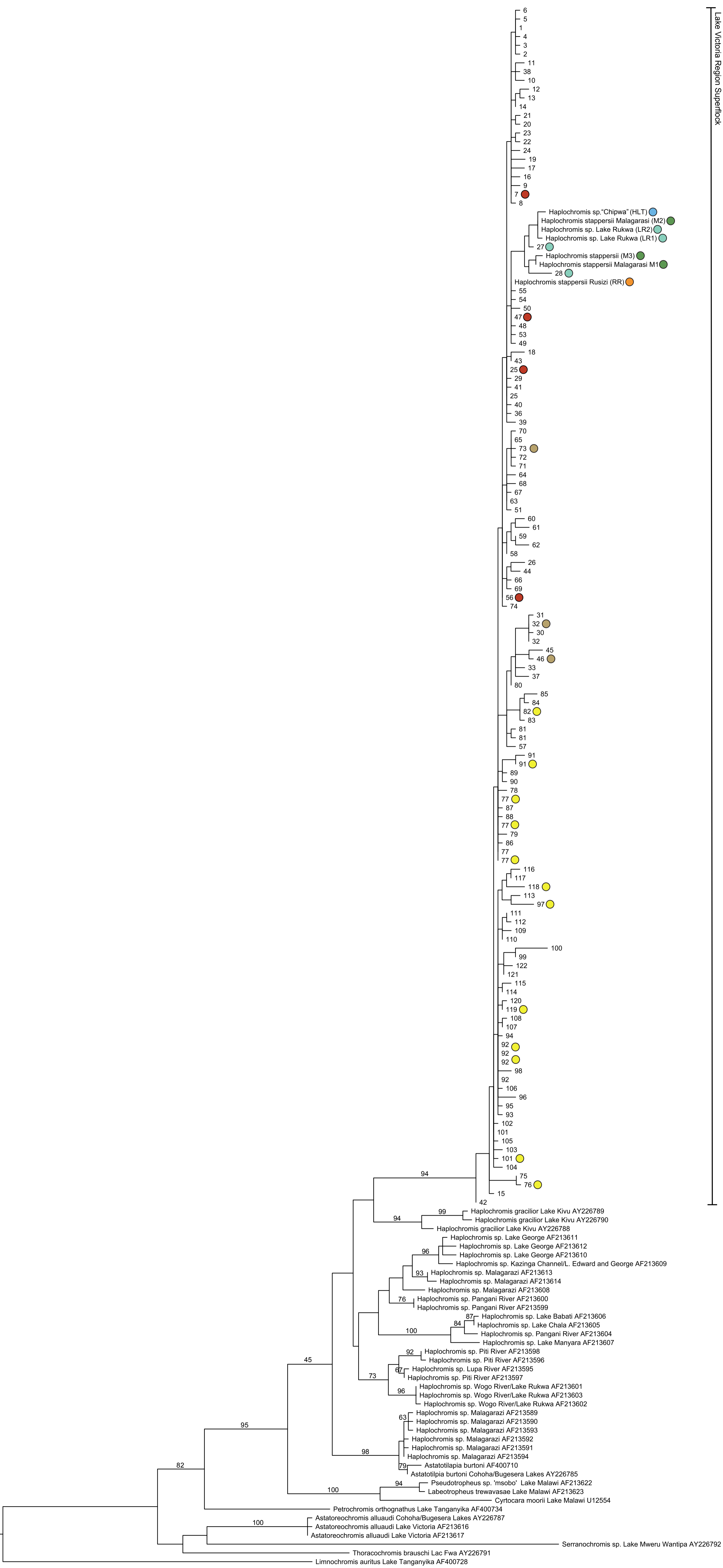

Supplement: S3.pdf [file rsos140498supp6.pdf]
